# Genetic properties of the MAGIC maize population: a new platform for high definition QTL mapping in *Zea mays*

**DOI:** 10.1186/s13059-015-0716-z

**Published:** 2015-09-11

**Authors:** Matteo Dell’Acqua, Daniel M. Gatti, Giorgio Pea, Federica Cattonaro, Frederik Coppens, Gabriele Magris, Aye L. Hlaing, Htay H. Aung, Hilde Nelissen, Joke Baute, Elisabetta Frascaroli, Gary A. Churchill, Dirk Inzé, Michele Morgante, Mario Enrico Pè

**Affiliations:** Institute of Life Sciences, Scuola Superiore Sant’Anna, Pisa, Italy; The Jackson Laboratory, Bar Harbor, Maine USA; Institute of Applied Genomics, Udine, Italy; Department of Plant Biotechnology and Bioinformatics, Ghent University, Gent, Belgium; Department of Agricultural and Environmental Sciences, University of Udine, Udine, Italy; Department of Plant Systems Biology, VIB, Gent, Belgium; Department of Agricultural Sciences, University of Bologna, Bologna, Italy; Current address: Thermo Fisher Scientific, Via G.B Tiepolo 18, 20900 Monza, MB Italy; Current address: Department of Agricultural Research, Nay Pyi Taw, Myanmar; Current address: Plant Biotechnology Center, Yangon, Myanmar

## Abstract

**Background:**

Maize (*Zea mays*) is a globally produced crop with broad genetic and phenotypic variation. New tools that improve our understanding of the genetic basis of quantitative traits are needed to guide predictive crop breeding. We have produced the first balanced multi-parental population in maize, a tool that provides high diversity and dense recombination events to allow routine quantitative trait loci (QTL) mapping in maize.

**Results:**

We produced 1,636 MAGIC maize recombinant inbred lines derived from eight genetically diverse founder lines. The characterization of 529 MAGIC maize lines shows that the population is a balanced, evenly differentiated mosaic of the eight founders, with mapping power and resolution strengthened by high minor allele frequencies and a fast decay of linkage disequilibrium. We show how MAGIC maize may find strong candidate genes by incorporating genome sequencing and transcriptomics data. We discuss three QTL for grain yield and three for flowering time, reporting candidate genes. Power simulations show that subsets of MAGIC maize might achieve high-power and high-definition QTL mapping.

**Conclusions:**

We demonstrate MAGIC maize’s value in identifying the genetic bases of complex traits of agronomic relevance. The design of MAGIC maize allows the accumulation of sequencing and transcriptomics layers to guide the identification of candidate genes for a number of maize traits at different developmental stages. The characterization of the full MAGIC maize population will lead to higher power and definition in QTL mapping, and lay the basis for improved understanding of maize phenotypes, heterosis included. MAGIC maize is available to researchers.

**Electronic supplementary material:**

The online version of this article (doi:10.1186/s13059-015-0716-z) contains supplementary material, which is available to authorized users.

## Background

Maize (*Zea mays* L.) is an important model organism and a global agricultural resource that exhibits enormous variation in quantitative traits. A better understanding of the genetic basis of quantitative variation in maize will improve predictive crop genetics. While high throughput DNA sequencing of individuals is becoming routine [1, 2], linking complex phenotypes to their molecular basis remains a major challenge. Genetic mapping is a powerful strategy that exploits genomic information to dissect complex traits into Mendelian loci (quantitative trait loci or QTL) and identifies genetic determinants that may lead to crop improvement. As marker density ceases to be a limiting factor [3], our ability to discover specific genetic determinants in a single mapping study depends upon the availability of populations with high genetic diversity and recombination density [4]. Linkage mapping in plants has traditionally used bi-parental crosses, in which two inbred founders are crossed to produce genetically segregating progeny. The progeny genomes are reconstructed from the founder haplotypes, and QTL are mapped by their association to genetic markers. Such populations provide high mapping power, but suffer from a shortage of diversity and recombination events. An alternative approach is association mapping on diversity panels, in which individuals with unknown kinship are selected. Association mapping benefits from high genetic diversity and a historical accumulation of recombination events, but its efficacy is limited by undetermined pedigrees and missing parental information.

Multi-parent cross designs (MpCD) bridge the two approaches and dramatically increase mapping resolution and power by incorporating greater genetic diversity and by increasing the number of crossing generations in elevated minor allele frequency (MAF). MpCD are produced by crossing more than two inbred founder lines in one of three ways: (1) by creating panels of recombinant inbred lines (RIL) that are mosaics of the founder genomes (for example, mouse Collaborative Cross (CC) [5] or Multi-parent Advanced Generation InterCrosses (MAGIC) populations [6–8]); (2) by breeding a single reference inbred line to many inbred lines and creating multiple bi-parent RIL (for example, Nested Association Mapping (NAM) panel [9], Dent and Flint panels [10]); or (3) by crossing *n* founders and maintaining an outbred population (for example, Diversity Outbred (DO) in mice [11, 12] and Heterogenous Stock (NIH-HS) in rat [13]). All of these designs produce mapping populations with superior genetic diversity [14], smaller haplotype blocks [15], and higher mapping power [16] than bi-parental mapping panels. The MAGIC, CC, and NAM designs produce a reusable reference population of RIL that can be genotyped once and phenotyped repeatedly, which reduces mapping costs and allows phenotypic data to be accumulated over time [17]. When the founder genomes have been fully sequenced, association mapping can be performed by imputing the founder sequences onto the MpCD genomes, which may provide single nucleotide mapping resolution [13]. Combined with founder expression data, these populations can lead to the discovery of variants associated with both expression and structural variation.

The power of MpCD has not been fully exploited in maize. A large NAM population has been produced [9], and collections of related bi-parental populations were also recently developed and applied to genome-based prediction in maize [10] and QTL mapping [18, 19]. However, the genetic variability in these panels is spread across bi-parental RIL families (25 with a common recurrent parent in the case of NAM), each with limited mapping power. Here we provide the first description of the MAGIC maize (MM) population, the first balanced MpCD developed in maize that integrates the diversity of eight diverse inbred founder lines into 1,636 RIL-F_6_ made available to researchers. The MM is a new mapping population that contains a large amount of genetic diversity and fine recombination block structure within a high MAF. We describe the genetic properties of the MM and evaluate its mapping power by simulating multiple QTL under varying MAF, sample sizes, and effect sizes. Finally, we test the MM population on field-collected phenotypes, suggesting the role of structural variation in grain yield, dissecting a complex QTL for flowering time, and discussing suggestive candidate genes for minor QTL. The MM is a powerful new tool that integrates the technological advances of the past decade to advance our understanding of the genetic basis of quantitative traits in maize. The MM lines are stocked at Scuola Superiore Sant’Anna (IT) and are available free of charge for research purposes.

## Results

### Composition and diversity of the MAGIC maize population

**Fig. 1:**
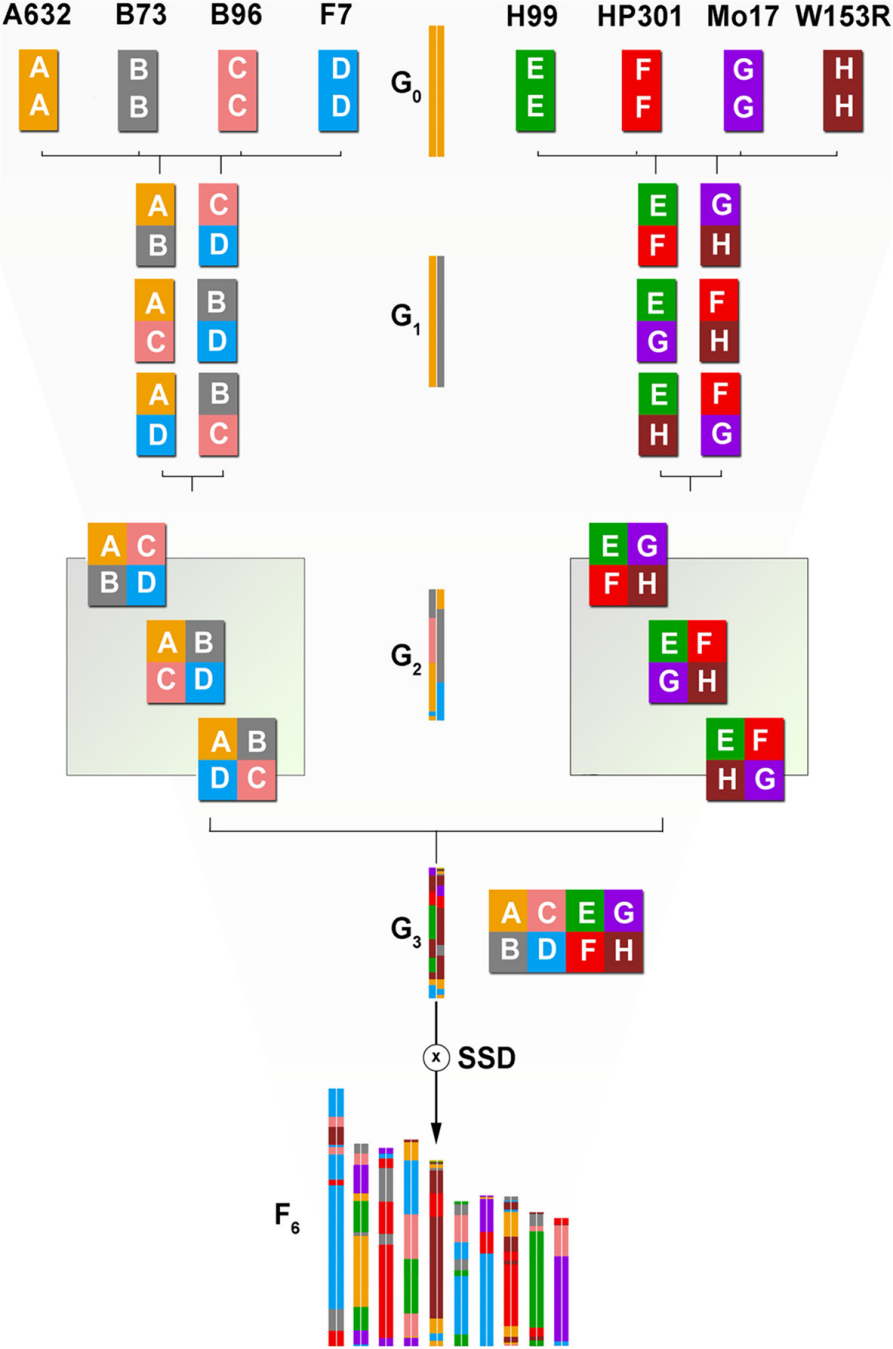
Breeding funnel of the MAGIC maize population. RIL were produced according to a funnel design with pooling to establish 35 subfamilies (one shown). Colors and letters refer to founders as indicated at the top of the image. Colored bars in the middle depict the composition of a single diploid chromosome throughout the left branch of the breeding funnel. At G_0_ eight maize inbred lines are crossed in a half-diallel design to all possible hybrids. The 28 two-way hybrids (G_1_) are permuted into different funnels, each producing an MM subfamily. The two-way hybrids with no founders in common are crossed for a total of 210 individual entries (G_2_) later pooled into 70 four-way collections having the same founder alleles in different *cis* combinations. Four-way pools are crossed with their complement, establishing the eight-way progenitors (G_3_). About 250 seeds from each eight-way progenitor are randomly chosen and sown establishing the MM lines. Each of at least 50 lines per subfamily is advanced through single seed descent (SSD) up to F_6_. The genomic composition based on haplotype reconstruction of MM line 35_5 is shown at the bottom

**Fig. 2:**
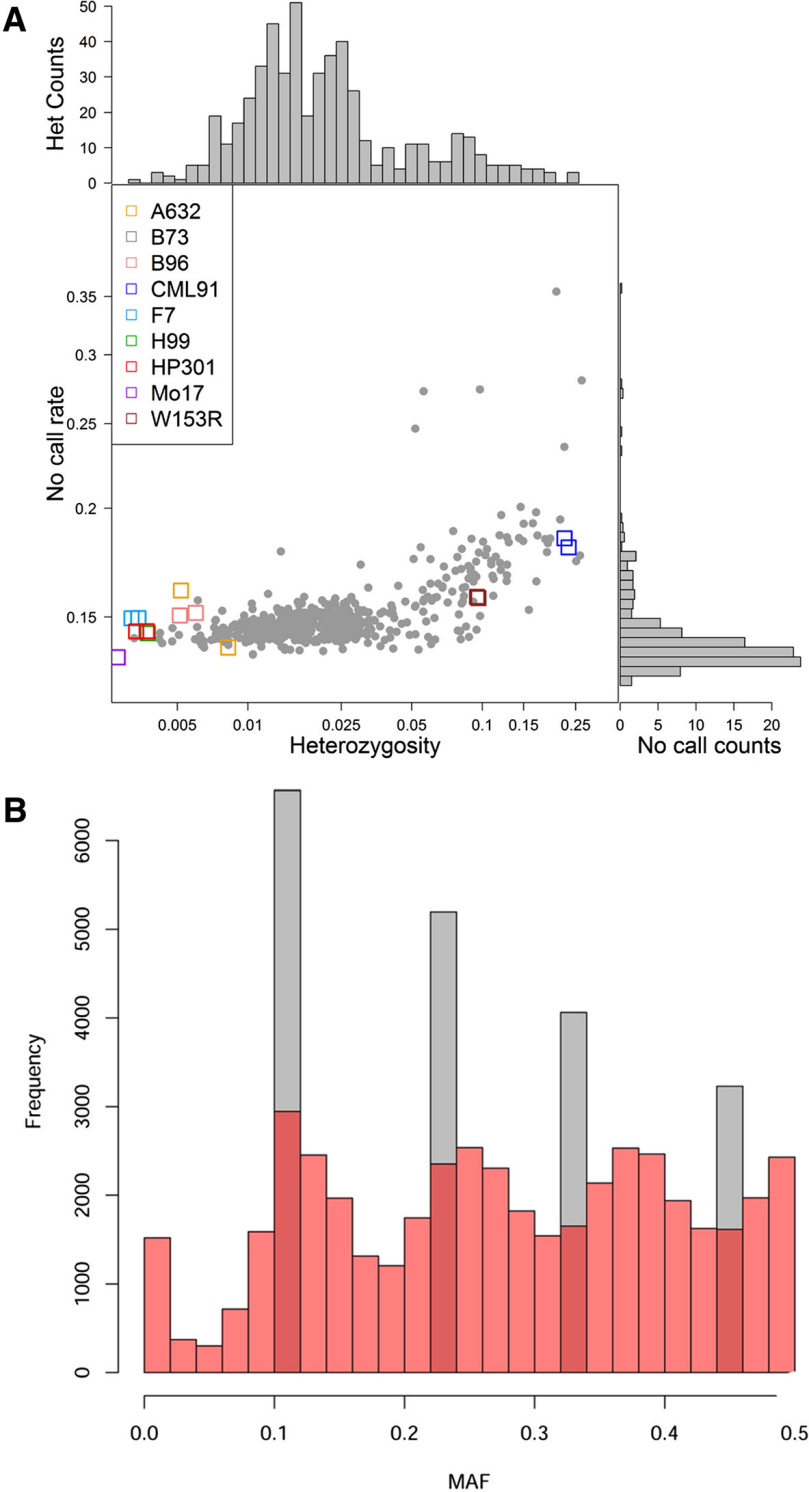
Genomic features of the MM population. In panel **a**, heterozygosity and allele call failure rate from the Illumina MaizeSNP50 BeadChip in the MM panel. The middle graph reports individual MM lines as gray dots and replicas of founder inbred lines as colored squares. CML91 (seldom present in the MM) and W153R have higher heterozygosity than expected. The other inbreds have residual heterozygosity close to 0.5%. The two replicas of B73, and one each for Mo17 and H99 are not visible because of exceedingly small heterozygosity (0.02%). The observed heterozygosity for the MM (top histogram; mean 3.43%; mode 1.4%) is skewed to the left. The MM lines showing high heterozygosity also show higher failure rate (right histogram), likely due to artifacts in allele calls. In panel **b**, MAF distribution in the MM founders (gray) and MM lines (red) on a subset of SNP fully genotyped and homozygous in the founder lines. MAF distribution in founders is skewed towards rare alleles, confirming founders’ diversity. This is partially contributed by residual heterozygosity. MAF 0.5 is not reached because nine founders are considered in this calculation. The breeding design successfully shuffled alleles, leveling MAF distribution around founders’ frequency classes

Eight maize inbred lines (A632, B73, B96, F7, H99, HP301, Mo17, W153R; referred to using letters A-H, respectively. Additional file 1: Table S1) were crossed in a funnel breeding design to produce 1,636 MM RIL-F_6_ (Fig. 1 and Additional file 2: Table S2). RIL lines were produced by pooling two-way, four-way, and eight-way hybrids in 35 independent breeding funnels (subfamilies) in the format [(AxB/CxD)+(AxC/BxD)+(AxD/BxC)]/[(ExF/GxH)+(ExG/FxH)+(ExH/FxG)]. A ninth parent (CLM91) was introduced as the two-way B73xCML91 hybrid 20 times in 15 subfamilies, to complement four-way crosses having B96xHP301 that failed. Each funnel was advanced by single seed descent (SSD) to the F_6_ generation. We genotyped the founder lines and 529 MM lines using the Illumina MaizeSNP50 BeadChip [20] retaining 54,234 SNPs that mapped to the RefGenV3 B73 genome [21]. The proportion of polymorphic alleles among founders varies from 0.29 (B73 vs. A632) to 0.48 (B73 vs. Mo17). Residual heterozygosity in seven of the founders was close to 0.5% or less, except for CML91 (22.4%) and W153R (9.5%). MM lines at F_6_ have an average heterozygosity of 3.43%, slightly higher than the expected value after SSD (3.125%). Mean call rate for the full SNP dataset was 85% (Fig. 2a). The minor allele frequency in the MM is generally between one-eighth and one-half (Fig. 2b). We did not observe a strong enrichment of residual heterozygosity in pericentromeric regions except on Chr 8; conversely, we observed higher heterozygosity in the telomeric regions of Chr 6 and 7 (Additional file 3: Figure S1). The distribution of observed heterozygosity is not related to that of polymorphism rate (Additional file 4: Figure S2). Local heterozygosity enrichments outside pericentromeric regions are apparent in some chromosomes, notably Chr 4, Chr 7, and Chr 10 (Additional file 4: Figure S2).

**Fig. 3:**
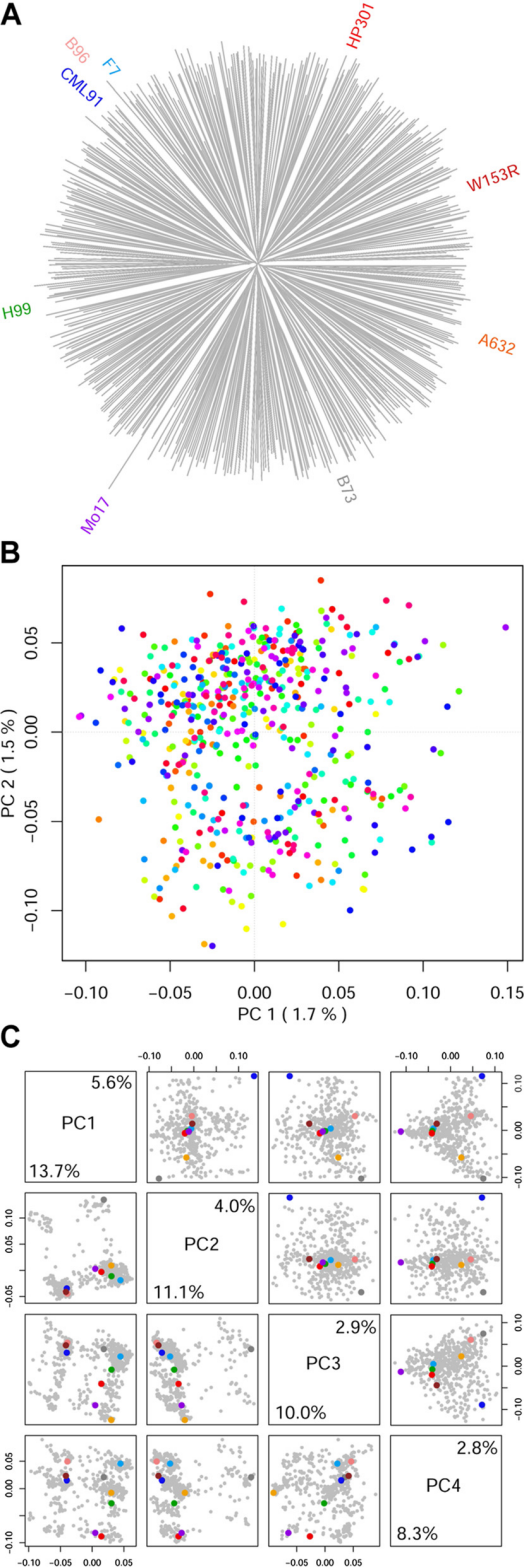
Diversity in the MM population. Panel **a** depicts a NJ phylogeny of the MM lines, with MM founder labels depicted radially. The long terminal branches and deep common ancestors show that MM lines are genetically diverse and unique to each other. Founder lines are also evenly distributed, except for Mo17, whose diversity is overestimated by the genotypic array design. Panel **b** shows a principal component (PC) analysis of the full set of SNPs. Different colors represent different subfamilies. The low PC loadings, reported on axes, confirm no structure in the dataset. In panel **c** the first four PC (PC 1–4) from a subset of centromeric (bottom left) and telomeric SNPs (top right) are shown. The relative PC loadings are shown along the diagonal, with values cornering either telomeric or centromeric regions. MM founders are color coded according to Fig. 3a. Pericentromeric regions still present a structure that is lost in the telomeric regions of the MM lines, as confirmed by higher PC loadings

**Fig. 4:**
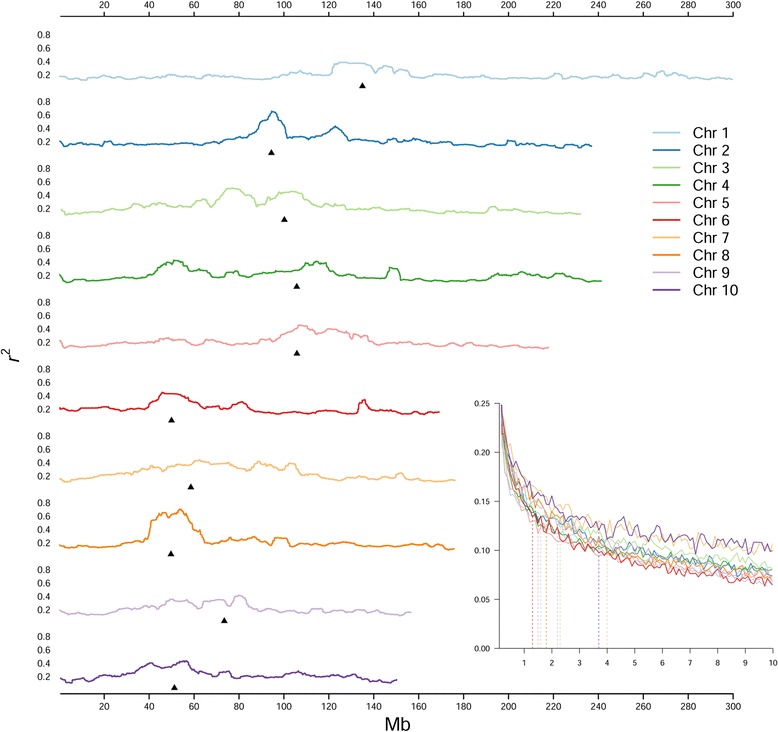
Genomic linkage disequilibrium (LD) in the MM population. The insert bottom right shows chromosome specific LD decay in the MM population with colors according to legend. Mean *r* ^*2*^ (y axis) is averaged in subsequent windows ad plotted on physical distance (x axis). LD is generally low and halves within 1 to 4 Mb (vertical dashed lines) suggesting high mapping resolution. Mean LD along chromosomes is plotted in the bigger panel. Each marker is considered separately, averaging for each the pairwise *r* ^*2*^ with all surrounding markers within ±1 Mb. Individual markers’ *r* ^*2*^ are then averaged in sliding windows of size 100 markers. Black arrows point centromeres. LD is generally higher in centromeric regions, but regions of higher LD are also present elsewhere (see Discussion in text)

We selected a set of 5,443 SNPs with low linkage disequilibrium (LD) (*r*^*2*^ ≤0.4) and produced a neighbor joining (NJ) phylogenetic tree (Fig. 3a, magnified in Additional file 5: Figure S3). We found that the MM lines are distributed at equal distances from the root of the tree. We performed principal component analysis (PCA) on the MM lines [22] and found that the first 10 PCs explain only 13% of the variance (Fig. 3b and Additional file 6: Figure S4). Some structure is still present in pericentromeric regions, as shown when comparing SNPs within ± 1 megabase (Mb) of centromeric regions with equivalent telomeric regions (Fig. 3c). We observed LD decay as a function of physical distance using mapped array SNPs. We found low LD baseline for all chromosomes and an LD halving distance between 1 and 4 Mb (Fig. 4, insert). The low LD in the MM population suggests it can achieve high mapping definition. Local LD pattern in the MM genomes was calculated considering the average *r*^*2*^ for each marker within a ±1 Mb window. This measure was chosen in accordance to an intermediate LD halving distance (Fig. 4, insert). This analysis confirms less recombination in pericentromeric regions, but shows blocks with higher LD outside centromeres (Fig. 4). The local pattern of the 25^th^ and 75^th^ percentiles of LD decay distribution are mostly in accordance (Additional file 7: Figure S5).

### MAGIC maize genomes

**Fig. 5:**
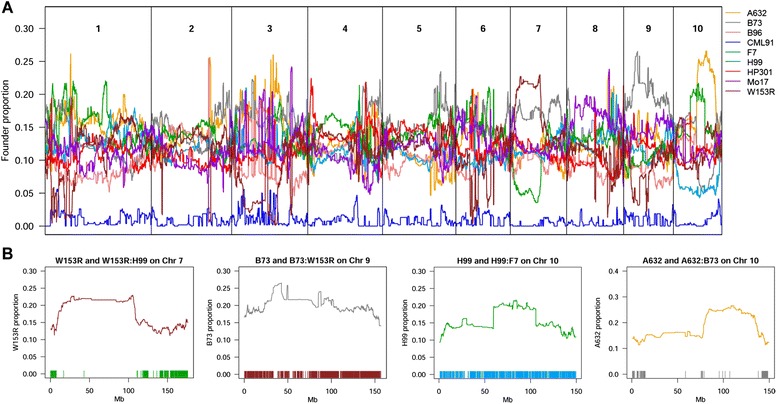
Locus-based founders contribution to the MM genomes. Panel **a** shows that founders’ contribution to MM lines genomes is close to 12.5%, except for CML91, seldom introduced. Because of this, CML91 was excluded from QTL analyses. Some regions still show significant deviation from the expected one-eighth. Regions of Chr 7, 9, and 10 in which founder proportions are distorted are shown in panel (**b**). Each line depicts the contribution of the founder that is over-represented. Below the line, each tick (colored according to legend in panel (a) indicates a polymorphism between the over-represented and the under-represented founder. The allelic distortion on Chr 7, 10 (for A632 vs. B73), and partially 9 can be related with IBS regions in which the haplotype model cannot distinguish between the two founders. The under-representation of F7 on Chr 10 has another cause (see text)

We derived the MM genetic map by anchoring the intermated B73 x Mo17 (IBM) genetic map positions [20] over 54,234 SNPs on RefGenV3 and interpolating missing centimorgan (cM) values proportional to physical distances between markers (Additional file 8: Table S3). We reconstructed the RIL genome mosaics in terms of the eight or nine founder haplotypes using a hidden Markov model (HMM) [12]. Because of the low residual heterozygosity detected in the MM (Fig. 2a), the hidden states were the eight (or nine) homozygous genotypes. MM lines deriving from eight parents were allowed to have eight genotype states, whereas those bearing CML91 haplotypes were allowed nine genotype states. Crossover probabilities between any two markers were calculated *r*(*4*-*r*)/(*1*+*2r*), as in the eight-way CC-like MpCD produced by selfing [23]. After haplotype reconstruction, the MM lines show on average 80.9 recombination events. Given the breeding design (Additional file 2: Table S2) the expected parental contribution to the overall MM population would have been 12.50% for A632, F7, H99, Mo17, and W153R, 10.71% for HP301 and B96, 14.29% for B73 and 1.79% for CML91. The observed contribution is biased towards the over-representation of A632 and H99 and the under-representation of W153R and B96 (Additional file 9: Figure S6). Low B96 representation may be due to unintentional selection against late flowering genotypes. The average founder contribution per locus (Fig. 5a) places around 12.5%, but several deviations can be observed. While some deviations may have biological causes (see Discussion), others are likely caused by marker selection bias on the genotyping platform limiting our ability to distinguish between some lines. This is the case on Chr 7, Chr 8, and partially Chr 9, where few SNPs distinguish the over-represented founder from the symmetrically under-represented founder (Fig. 5b). F7 has a lower contribution on Chr 10 that is currently unexplained by either low diversity regions or biological causes. A flowering time QTL was reported on chr 10 having *ZmCCT* as causal gene [24], which might have contributed to local distortion on the contribution of the early flowering F7.

### MAGIC maize founders sequencing and transcriptomics data

The ability to identify candidate genes in MAGIC maize QTL mapping is improved by incorporating whole genome sequencing and transcriptomics data of the founder lines. Whole genome sequencing of A632, F7, H99, HP301, and W153R produced a total of 3,130,725,650 paired-end reads with short inserts, ranging in median size from 187.35 to 422.12 bp (*σ* from 15.24 to 117.18 bp). The coverage ranged from 20.6x (F7) to 33.22x (A632) (Additional file 10: Table S4). By combining the previously sequenced genomes of Mo17 and B73 to those we produced, we obtained a set of 27,752,155 SNPs to be used in association mapping by imputing founder SNPs onto the reconstructed haplotypes of the MM lines. We also performed RNA sequencing on the fourth leaf stage of A632, B73, F7, H99, HP301, Mo17, W153R, and CML91 producing on average 27 million raw reads each.

### MAGIC maize power simulation

**Fig. 6:**
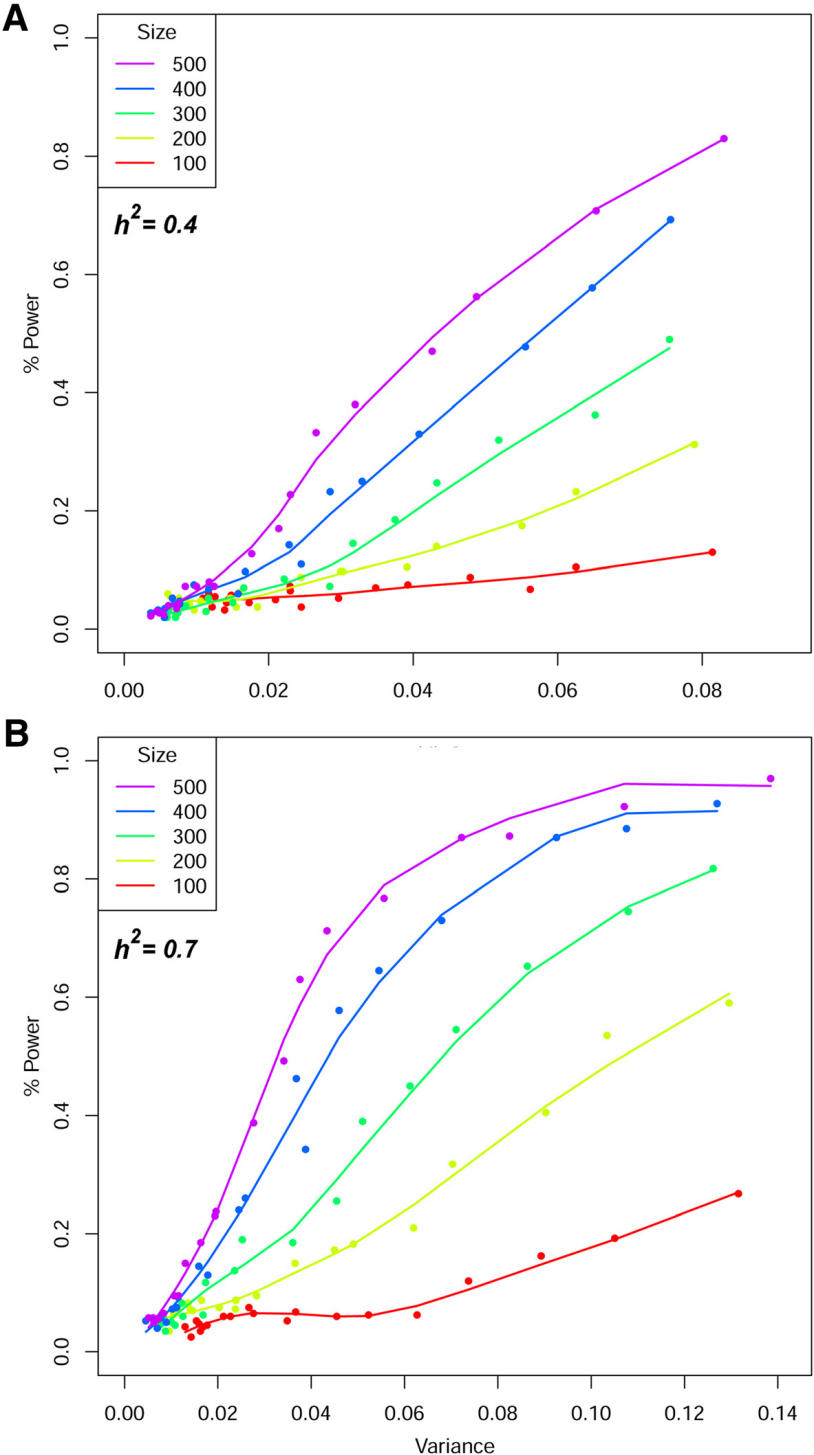
Power simulations on the MAGIC maize. Average power values for 400 independent runs (100 for each of MAF 0.125, 0.25, 0.375, and 0.5) are given as a function of the variance explained by one of 20 QTL simulated with effects following a geometric series. Panel **a** reports the case with *h* ^*2*^= 0.4, panel **b** with *h* ^*2*^= 0.7. Power is influenced by sample size

We performed power simulations to estimate the number of MM lines required to detect QTL of a certain effect size. We simulated 20 QTL with effects following a geometric series with the same principle that drove simulations on the NAM population [25], under heritability of either 0.4 (Fig. 6a) or 0.7 (Fig. 6b). We found that power increased with increasing sample size (from 100 to 500), and increasing effect size. Note that mapping resolution also rises with sample size as more lines increase the number of observed recombination events. At heritability 0.7, the use of 500 samples permits the detection of QTL explaining 8% phenotypic variance with >90% power (Additional file 11: Table S5). QTL mapping with 100 MM lines is far from this power, yet panels as small as 300 already mirror the QTL mapping with 500 MM lines. Three hundred MM lines detect QTL accounting 12% of variance with a power of 82%. Sample sizes of 500 and 400 approach a plateau of high power with QTL explaining about 10% of variance. The same simulations were plotted with power as a function of sample size alone to allow a graphical comparison with the NAM power report [25] (Additional file 12: Figure S7). QTL were sorted in effect size quartiles to survey the MM mapping power with high-effect and low-effect QTL separately. Using 500 MM lines with heritability 0.7 permits to detect more than 40% of the 20 simulated QTL. In this scenario, the five QTL having the highest effect are detected with a power close to 90%, whilst QTL with a lower effect are hardly identified.

### QTL mapping with the MAGIC maize

The MAGIC maize mapping power was experimentally assessed by planting 529 MM lines (at least 15 lines per subfamily) in two different environments and measuring days to pollen shed (PS), plant height (PH), ear height (EH), and grain yield (GY) (Additional file 13: Table S6). As expected, the wide genetic diversity in the MM produced broad phenotypic variation for all four traits. We observed a smooth distribution of values around the mean in all traits (Additional file 14: Figure S8 and Additional file 15: Table S7). We reconstructed the founder haplotypes in the MM and performed linkage mapping of PS, PH, EH, and GY [26, 27]. We estimated significance thresholds by permutation to determine significant peaks (*P* <0.01). After the initial genome scan, we repeated QTL analysis for each trait by including the major QTL as a covariate to the model (Additional files 16, 17, 18, and 19: Figures S9–S12). Peaks above the suggestive logarithm of odds (LOD) score (*P* <0.63) [28] are reported in Additional file 20: Table S8. Here we describe the identification of QTL candidates using founder sequencing and transcriptomics data.

**Fig. 7:**
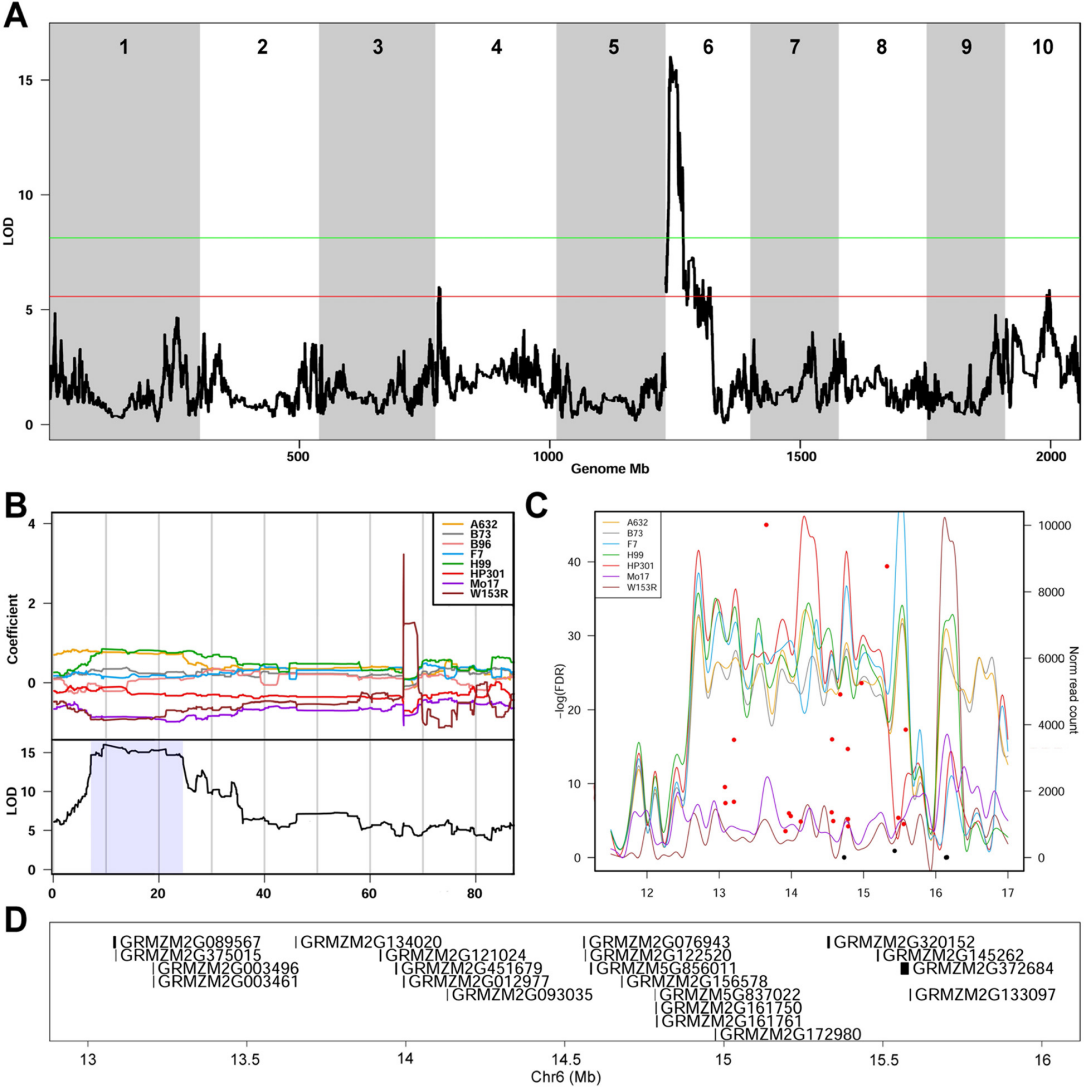
Dissection of a major QTL for GY. In panel **a** GY shows a major QTL on the short arm of Chr 6 (pleiotropic on PH and EH). Green line and red line represent strong (*P* <0.01) and suggestive (*P* <0.63) thresholds. Panel **b** magnifies the QTL region and shows that the QTL has a flat and wide top, suggesting the involvement of a series of *cis* causal variants. The founder effect plot shows that at this locus the W153R and Mo17 alleles are associated with low GY. Panel **c** shows that 21 genes have differential expression between these and the remaining founders in a region spanning 2.5 Mb (red dots; black dots for not significant differential expression tests). Also in panel **c**, normalized sequencing read counts of founders in 0.1 Mb bins in the same region reveal substantially less reads for Mo17 and W153R, confirming the extant SV. Note that downstream this region the read counts for Mo17 and W153R are similar to that of other founders, ceasing the differential expression. In panel **d** are reported genes differentially expressed in the SV region

We identified three suggestive QTL for GY (Additional file 20: Table S8 and Additional file 16: Figure S9). Using expression data from the founder lines, we searched genes in QTL intervals having differential expression (FDR <0.05) matching founder allele effects estimated by the mapping model. Overall, we identified 45 such genes for GY QTL (Additional file 21: Table S9). The major QTL for GY is a locus on the short arm of Chr 6 pleiotropic to PH and EH (Fig. 7a, Additional file 20: Table S8, Additional files 17 and 18: Figure S10 and S11), and accounting for 13% of the variance in GY. The founder allele effects at the locus show that Mo17 and W153R alleles contribute to low GY values (Fig. 7b, top panel). The corresponding LOD curve draws a plateau of high significance spanning 17.4 Mb (Fig. 7b, bottom panel), encompassing 24 differentially expressed genes (FDR <0.001) between Mo17 and W153R and the remaining founders. Twenty-one of these differentially expressed genes are located in a 2.5 Mb interval. The analysis of the MM founders’ genomic sequences in this interval revealed a region of structural variation (SV) in which reads from Mo17 and W153R were absent (Fig. 7c and d). This suggests that SV in Mo17 and W153R may be responsible for this GY QTL in the MM. The analysis of minor QTL for GY also led to interesting candidates. *GRMZM2G054651* is one of four differentially expressed genes within a smaller QTL for GY on Chr 4 (5.3-10 Mb), where HP301 bears the low allele. According to Plaza 3.0 [29] this gene encodes for a HVA22-like protein. *HVA22* is a gene originally cloned from barley [30] and involved in hormonal response to ABA. *GRMZM2G101875* (FDR <0.001) is one of five differentially expressed genes associated with the GY QTL on Chr 10 (78.4-95 Mb). This gene has its best ortholog in *CER8*, an *Arabidopsis* gene encoding a chain acyl-CoA synthetase.

**Fig. 8:**
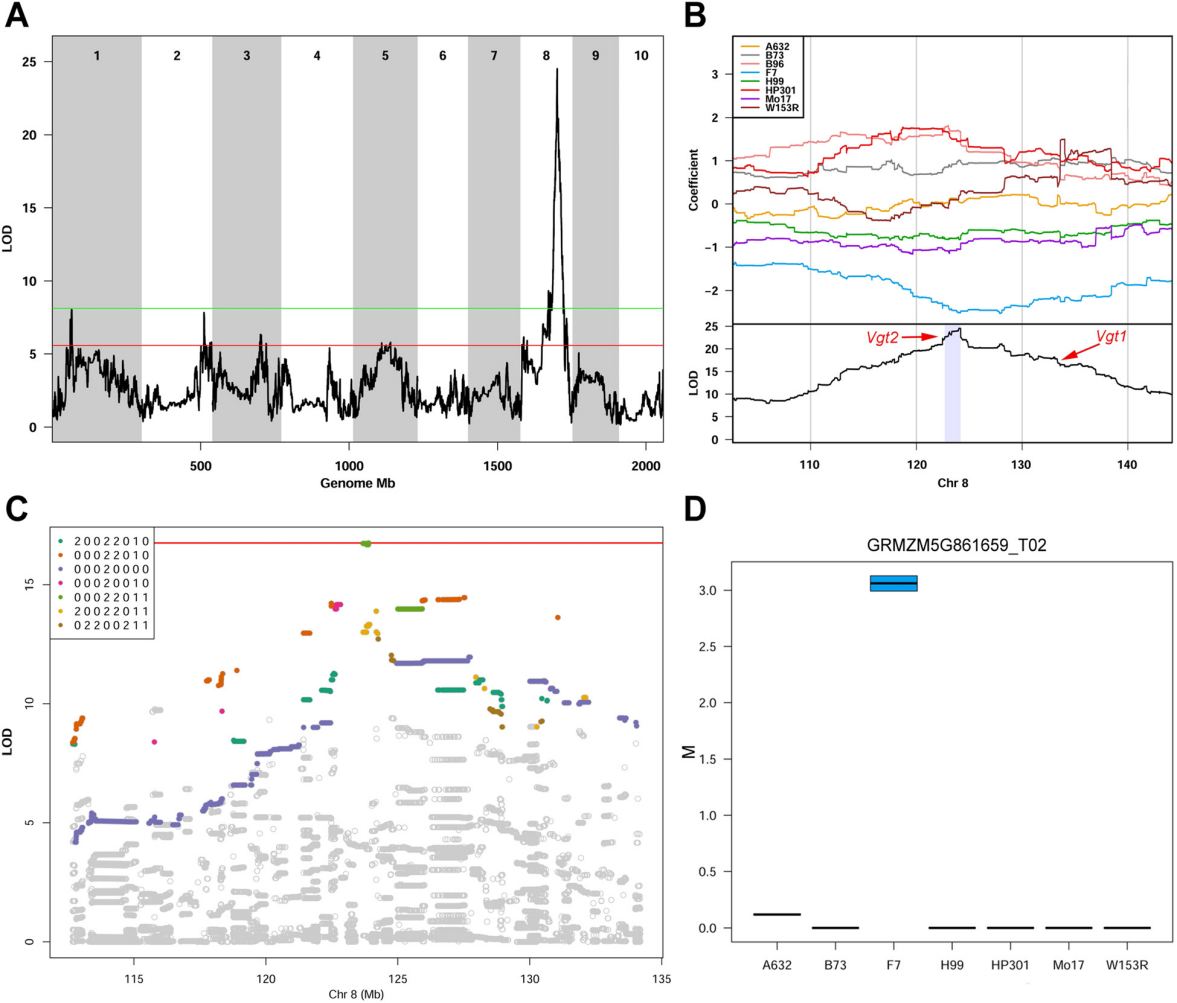
Fine mapping of a major QTL for PS. The linkage mapping approach shows a major QTL contributing to PS on Chr 8 (panel **a**). Green line and red line represent strong (*P* <0.01) and suggestive (*P* <0.63) thresholds. The top part of panel **b** shows founders coefficients. F7 (early flowering) contributes with low alleles to the QTL. The bottom part shows the LOD curve, red arrows point out *Vgt2* and *Vgt1* loci. Gray shading identifies the confidence interval of the QTL. Note that the region is magnified to show 50 Mb only. In panel **c** the outcome of the association mapping in the QTL area is reported. The red line is the threshold for significant associations after 500 permutations (*P* <0.01). Gray dots represent imputed SNPs, and colors are given to common haplotypes. In legend, numbers are given for founder imputed alleles (0 for reference, 1 for heterozygous, 2 for alternative). Haplotype strings indicate founders according to the A-H order. No founder besides the early flowering F7 show a private haplotype in the region. Panel **d** shows the expression pattern of *GRMZM5G861659*, within 1 Mb downstream the QTL confidence interval and matching founders’ contribution to the QTL

We found several suggestive QTL loci for PS in the MM (Additional file 20: Table S8 and Additional file 19: Figure S12). The analysis of differential expression within these QTL identified 101 genes matching founders’ contribution at FDR <0.05 (Additional file 22: Table S10). We found a pleiotropic QTL on Chr 8 explaining 19% of flowering time variance and having effects on PH and EH (Fig. 8a, Additional file 20: Table S8, Additional files 17 and 18: Figures S10 and S11). The pattern of founder effects splits into three groups, suggesting that there is either a single tri-allelic variant locus or at least two bi-allelic variant loci underlying the QTL (Fig. 8b, top panel). The F7 allele contributes to early flowering, while the B96 and HP301 alleles to late flowering. Previous studies have also found a QTL for PS in the same region on Chr 8 [31, 32], and the *Vgt1* locus at 132 Mb is a major QTL for flowering time cloned in maize [33]. The maximum LOD score for our PS QTL occurs at 124.0182 Mb; a 2 LOD drop support interval spans a 1.5 Mb region which overlaps *Vgt2*, a major locus involved in flowering time [34] (Fig. 8b, bottom panel). The lead candidate gene for *Vgt2* is *ZCN8*, a floral activator involved in photoperiod sensitivity [35] repeatedly identified by studies on diversity panels and inbred lines collections [19, 36, 37]. *ZCN8* is included in the confidence interval of the QTL we identified. Using the MM haplotype reconstructions, we imputed the founder sequences onto the MM genomes and performed association mapping in the QTL interval. This led to the identification of a haplotype 211 Kb long (123,682,690 to 123,893,776; *P* <0.05) (Fig. 8c), 500 Kb upstream the reverse-stranded *ZCN8*. In the recent update of the maize genome annotation (RefGenV3) *ZCN8* was moved from 123.5 to 123.0 Mb. Within this highly significant 211 Kb haplotype, five newly characterized pre long-non-coding RNAs, possibly precursors of small RNA [38], are present. LD between imputed SNPs within the QTL confidence interval (3,760) is low, with few linkage blocks visible (Additional file 23: Figure S13). In the ±1 Mb region beyond the confidence interval we identified *GRMZM5G861659*, the only gene with expression matching the founder effects. This gene encodes a POZ and MATH domain containing protein and is only expressed in the very early flowering F7 background (Fig. 8d). Other suggestive QTL with smaller effects on PS were identified. The QTL on Chr 1 at 156.5 Mb shows differential expression of *GRMZM2G429759* (FDR <10^−6^), which have sequence similarity with the *BRASSINOSTEROID INSENSITIVE 1*-associated receptor kinase 1 in *Oryza sativa* spp. *indica* (75.68% BLAST identity, *e*-*value* = 9e-109). In the QTL on Chr 5 (126.3 Mb) the differentially expressed *GRMZM2G090480* has its *Arabidopsis* best ortholog in *MED18*, which encodes a subunit of the MEDIATOR complex, shown to affect different plant functions including flowering time [39].

## Discussion

### The genomes of MAGIC maize

The MM brings together high genetic diversity and low population structure in elevated MAF, all positive characteristics for QTL mapping [4]. This is the result of a breeding scheme that largely avoids directional selection during the production of the RIL. During production, the population was kept as large as possible, both to avoid genetic drift and to gather a large number of recombination events without the need of additional intermating generations. Although high level of sequence variation between the lines might inflate heterozygosity by inefficient hybridization on the chip, we did not observe significant departure from the values expected in the MM lines. We did not observe higher heterozygosity in centromeric regions [40], indicating that it was not selected during MM breeding [9]. The low observed pericentromeric heterozygosity might also have resulted from low marker resolution on the genotyping array in that region (Additional file 4: Figure S2). Keeping subfamilies separated allowed us to track the origin of each line once the final population was produced. The genetic distance between lines is evenly distributed (Fig. 3), which implies that subsets of the MM panel might be selected for specific research purposes without losing the general features of the population. The divergence of Mo17 from the other lines may be due to marker selection bias on the genotyping array, as B73 and Mo17 were used to select many of the SNPs on the genotyping array [20, 41]. This might also explain the apparent similarity between B96, CML91, and F7.

The LD extent is uniform across the genome (Fig. 4), some telomeric regions having slightly higher LD. While large-scale selection was avoided, selection on specific loci might have caused increased LD in bordering regions. On the short arm of Chr 4 (Fig. 4) this may be due to one of the founders, HP301, bearing the strong allele of *Gametophyte factor 1* (*Ga1*-*S*). *Ga1*-*S* is selected over *ga1* as *Ga1*-*S* hinders pollination from all the other inbred lines bearing *ga1* pollen [9, 42]. Selection for the allele likely inflates LD in the region, and this is confirmed by higher contribution from HP301 than of other founders in this region (Fig. 5a). Other regions of higher LD likely reflect the relationships between MM founder strains. Low diversity regions might in fact result in higher LD and in founder contribution distortion. Notably, the narrow LD peak at 150 Mb on Chr 4 (Fig. 4) corresponds to a CML91 contribution peak (Fig. 5a). The 25^th^ and 75^th^ percentiles of LD distribution across chromosomes are mostly in agreement, besides specific regions on Chr 2 and 6 (Additional file 7: Figure S5). These regions may be the remnants of high LD regions between the founders that were not efficiently broken by recombination during the breeding process.

After haplotype reconstruction, the MM lines showed an average of 80.9 recombination events (Additional file 9: Figure S6). The current MM genetic map is derived from the maize IBM population, whose two founders are also included in the MM. Its length (1,996 cM) can be used as a fair approximation to calculate the number of expected recombination events in the MM lines. In RILs, we sample one set of chromosomes and thus we count one round of recombinations per generation. We would observe no recombinations in G_0_. Assuming a maize genome length of 19.96 Morgans, we would observe 19.96 recombinations in G_1_ generation and likewise in G_2_ and in the first G_3_ selfing. One additional fully effective round of recombinations results from the single seed descent because of heterozygosity halving at each generation, bringing the total to 19.96 × 4 = 79.8. The observed recombinations in the MM (80.9) are thus remarkably close to the expectancy. Based on this count, we would expect more than 130,000 recombinations in the full population of 1,636 MM lines. Genome reconstruction, however, might be further improved by using sequence-based molecular markers. Work in other MpCD confirms that the genotyping approach may affect genome reconstruction efficacy [12], notably in the presence of wide regions identical by state (IBS). Deviations in the estimation of founder contributions in MM (Fig. 5) are likely due to the inability of the current genotyping method to distinguish between the founder lines. A632 and B73 are the most similar (Fig. 3a and Additional file 9: Figure S6), and this is expected since A632 and B73 were independently derived from the same source [43]. In the future, we envision low density sequencing approaches on the whole MM population that should allow us a finer reconstruction of RIL haplotypes by distinguishing between pairs of strains in IBS regions.

### QTL mapping with the MAGIC maize

The MM population represents a new and powerful tool for the fine dissection of quantitative traits in maize. Multi-parent crosses are the future of complex trait genetics [4]: here we have shown that the MM population contains roughly equal proportions of the founder genomes, that the genetic distance between the lines is evenly distributed and that the LD decays sharply. QTL mapping panels suffer a tradeoff between mapping power and definition. Faster LD decay increases the number of independently tested markers, which reduces power. However, the high MAF in the MM rescues the power to map rare variants. Simulation results showed that relatively small sample sizes could achieve high power for QTL detection (Fig. 6 and Additional file 12: Figure S7). Such results might be used as a guideline for choosing appropriate sample sizes for future studies. As the MM panel contains no population structure (Fig. 3b), any MM subsample may be used for QTL mapping. In contrast, fragmented, star-like designs, such as the maize NAM, require a higher number of samples to achieve effective QTL mapping.

The simultaneous simulation of several QTL reflects the genetic architecture of complex traits in maize, which is expected to be contributed by manifold QTL with medium to small effects [31]. We simulated 20 QTL on the MM to permit a comparison with the NAM panel, showing how a relative small number of MM lines can achieve high mapping power. One-third of the complete MM population confidently detects QTL with mid-to-high simulated effects, as our field test further showed. NAM simulations are not reported for less than 1,000 lines, yet a preliminary comparison with 500 MM lines provides interesting insights. In fact, under the same conditions of 20 simulated QTL with *h*^*2*^ = 0.7, 1,000 phenotyped NAM lines have an average mapping power of around 50%, while an MM panel half that size reaches a 41% power (500 lines; Additional file 9: Figure S6b). In the case of lower heritability (*h*^*2*^ =0.4; Additional file 12: Figure S7a), the MM power at 500 lines (22.1%) is also similar as that of twice the number of NAM lines. False positives appear higher in the MM than in the NAM, especially when exceedingly small number of lines are considered. The FDR trend in the MM however becomes rapidly lower, especially in the case of *h*^*2*^ = 0.7. Notably, the MM shows top-quartile QTL detection power higher than the NAM in both heritability conditions. This suggests that high effect QTL can be detected with small sample sizes. Finally, it is worth noting that each individual RIL of the MM encompasses more recombination events than a NAM RIL, increasing mapping definition when considering mapping panels of equal size. Once the genotyping of the whole MAGIC maize population is achieved, the power of the MM will be assessed in full.

The NAM and MM, as any artificial mapping panel, harness a subset of the genetic variation potentially available in a target species. The MM contains less diversity than the 25-founders NAM, yet it may provide higher mapping power in smaller numbers. This is expected since the MM puts more founder haplotypes into play in each individual RIL. In this study, we employed less than one-third of the full population and characterized a limited number of phenotypes on two fields to provide a demonstration of the MM mapping power. We thus focused on QTL with large effects, successfully mapping both known and novel loci. QTL mapping methods are an active area of research, and forward stepwise regression models are increasingly employed on different populations, NAM included [44]. Such methods provide best performances using elevated number of samples, allowing several rounds of regression to characterize complex traits architecture. However, as QTL effects become smaller, especially in conditions of low heritability, false positive rates may become relevant and hamper a reliable description of QTL. Because of this, in this preliminary mapping study we decided to limit to two rounds of mapping so to focus and discuss the QTL with largest effects. The comparison and selection of optimal mapping methods is a ceaseless work, and we foresee stepwise selection methods applied to the MM once more individuals are tested for phenotypes of interest. Since now, however, we showed that the MM might fit the needs of smaller research groups unable to manage massive experimental fields. This is especially relevant in experiments requiring phenotyping in controlled conditions, which hardly accommodate lines in the number of thousands. When QTL are discovered in the MAGIC maize population, it may be possible to perform validation studies using the broader variation of NAM lines and additional bi-parental panels between the MAGIC maize founders. Maize QTL mapping will benefit from the complementary use of the NAM and MAGIC maize populations for the gene-level dissection of quantitative traits.

We mapped a major QTL for GY that co-localizes with structural variation. Other researchers have found QTL in the same region of Chr 6 for maize GY [45–51] and kernel number per ear [48, 50, 51]. These QTL were found in different bi-parental mapping populations, for example, B73 x Mo17 [49], European flint F2 x Iodent [46], B73 x H99 [50, 51], and Chinese inbred genotypes Huangzao4 x Ye107 [47]. Recently, a nested bi-parental population in maize reported QTL for kernel weight and kernel maximum water content on the short arm of Chr 6 [18]. Structural variation is an extensive phenomenon in the maize genome, and nearby regions on Chr 6 contain SV influencing phenotypes [52, 53]. In tomato, an SV in a regulatory region was found to control fruit size through carpel number [54]. Three independent datasets (RIL genotypes, transcriptomics, and sequencing coverage) converged in the MM to suggest that this SV may contribute to GY. Further studies focusing on this 2.5 Mb region should permit to dissect the role of this region in GY. We speculate on other candidate genes involved in GY as identified by the convergence of independent methods on smaller QTL. Grain yield may be contributed by *GRMZM2G054651*, an HVA22-like protein. The RNA interference of *AtHVA22d*, one of *HVA22* homologs in *Arabidopsis*, caused smaller siliques and reduced yield [55]. A similar role in maize might be therefore hypothesized. *GRMZM2G101875*, with a highly significant differential expression test (FDR <0.001), has its best ortholog in *Arabidopsis CER8* gene. *CER8* was previously identified underneath a QTL for seed oil synthesis [56], a component of GY, thus supporting a similar role in maize. Although the QTL on Chr 6 has the highest effect, these candidates possibly contribute to GY as well. These QTL regions and candidate genes are effective starting points to unravel such a complex and important trait as grain yield. Additional studies with the clear intent of exploring yield and yield components using the power of the full MM might provide a crucial contribution in eventually deciphering the genetic background of grain yield in maize.

Our study confirmed that flowering time variation is the result of the cumulative effect of several small QTL, as first shown in the NAM [31]. The use of the 5,000 RIL in the NAM population identified altogether 36 QTL for days to anthesis. Of these, the MM confirmed seven loci within 5 Mb of the NAM association while identifying 22 novel loci above the *P* <0.63 threshold [28]. As the mapping simulation showed, QTL with small effects may be hard to detect with the current mapping method of the MM. This may contribute to the differences in QTL reported by the two populations. It should be noted that the number of RIL on which PS was mapped is 10-fold lower in the MM than in the NAM. The different results provided by the two populations is likely contributed by several other reasons, including: (1) different environments tested; (2) different statistical methods employed; and (3) different composition of the two panels (which share B73 and HP301 haplotypes only). *Vgt1* and *Vgt2*, the leading loci for maize flowering time, differ both in source breeding material and effects on flowering time [57]: we expect the same to stand for other PS loci and contribute to discrepancies between mapping populations. Our linkage mapping identified *ZCN8*, the main candidate gene for *Vgt2*, yet neither association mapping nor differential expression directly targeted *ZCN8*. The LD features of the QTL confidence interval calculated upon full genome imputation indicate a linkage peak right upstream *ZCN8*, among a generally low LD baseline (Additional file 23: Figure S13). B96, the latest flowering MM founder, does not contribute with its genome sequence to the genomic imputation, and possibly because of this the MM founders show no SNPs within this gene. *ZCN8* is not expressed at the fourth leaf stage from which our transcriptomics data were produced. However, we identified a genomic region that may have a *cis* regulatory role on *ZCN8*, as well as candidate genes possibly contributing to flowering time. Our results indicate that in addition to *ZCN8*, nearby regions might contribute to *Vgt2* locus. Association studies in both rice [58] and *Arabidopsis* [59] indicated that clusters of several linked elements might contribute to QTL regions. At a larger scale, this happens in maize with *Vgt1* and *Vgt2* [37], but this may hold true even within these QTL. This is not surprising, as also *Vgt1* was found to be regulated by noncoding elements acting on *ZmRap2.7* [60]. Possibly owing to this complexity, the MM did not identify a single causal variant for this locus, but rather pointed to several suggestive variants which may contribute to PS either independently or through *ZCN8*. In particular, the presence of five recently described pre long-non-coding RNAs in the region identified by our association mapping (*P* <0.05; Fig. 8c) urges to evaluate their role in maize PS. Non-coding RNA are involved in the control of numerous molecular mechanisms [61], including the regulation of the floral repressor gene *FLOWERING LOCUS C* (*FLC*) in *Arabidopsis* [62, 63]. Once the full genome sequencing of the eight MM founders will be completed and layers of targeted transcriptomics data added, this QTL will be dissected in full. PS in the MM is contributed by several other QTL with smaller effects, some of which providing interesting candidates. In the case of the QTL on Chr 5, the differentially expressed *GRMZM2G090480* has its best ortholog in *Arabidopsis MED18*, known to play a major role in flower organ formation and flowering time determination by up-regulating *FLC* and downregulating *AGAMOUS* (*AG*) [64]. For the QTL on Chr 1, *GRMZM2G429759* sequence similarity suggests a relation to brassinosteroid signaling. The role of brassinosteroid signaling on flowering time is well known in *Arabidopsis* [65, 66]. The high significance of its differential expression test (FDR <10^−6^) reinforces this candidate gene. Still our findings are only suggestive and further studies are required for a complete characterization of these predicted genes in the frame of flowering time pathway.

## Conclusions

The objective of this work was to assess the QTL mapping capacity of the MM rather than to provide a thorough QTL analysis. To do that, a deeper phenotypic characterization of the MM is required. Larger sample sizes (to a maximum of 1,696) will also provide greater resolution and power. Still, the number of suggestive candidate genes for two important and complex traits such as grain yield and flowering time identified by using only one-third of the MM is remarkable. Their hypothesized role in maize is enforced by the integrative orthology search carried out in Plaza 3.0 which, being based on four methods (BLAST-, clustering-, tree-, and collinearity-based) allows the projection of high-quality functional annotation over great phylogenetic distances [29]. The transcriptomics data currently used were limited to the fourth leaf stage. Previous studies demonstrated that transcript variation in leaves can be successfully correlated with trait variation at later stages [67–69], since *cis* allelic variants may affect expression. However, it is likely that adding layers of targeted transcriptomics data will further empower the ability of the MM to identify the causal variants of complex traits by targeting specific developmental stages. The full sequencing of all MM founders will also lead to a finer localization of QTL signals.

Mapping models for MAGIC populations are an active area of research [70, 71], and we anticipate that stepwise regression models, mixed models, adjustments for kinship, and Bayesian methods may improve mapping in the MM. Current mapping methods test one locus at a time, and development of multi-locus mapping methods in multi-parent populations is expected. Like other genetic reference populations, MM lines can be genotyped once and phenotyped repeatedly, allowing additional layers of transcriptional, proteomic, and metabolomic data to be accumulated on each line. MM lines can be phenotyped in multiple environments, which will increase our understanding of gene-environment interactions. The accumulation of such data will benefit from the MM being available to collaborators worldwide. The MM lines can also serve as the foundation for other crosses. Specific genes may be knocked in or out of MM lines to study the effect of genetic background on resulting phenotypes [72]. The MM lines might also be used to produce an outbred mapping population to increase the number of recombinations per sample and map QTL with even finer resolution. Finally, MM lines may be crossed to create up to 1,337,430 genetically distinct recombinant inbred intercrosses (RIX) [73]. These lines will not require genotyping because both parental chromosomes will have already been genotyped. Together, an outbred population and MM RIX would create unprecedented tools for the study of heterosis in maize.

## Materials and methods

### MAGIC maize breeding

The breeding scheme of the MAGIC maize is depicted in Fig. 1. In 2005 we choose eight inbred lines (IL) maximizing maize diversity [74] and ensuring germinability, viability, and reproducibility as founders of the MAGIC maize (G_0_): A632, B73, B96, F7, H99, HP301, Mo17, W153R (denoted by A to H, respectively). G_1_ (two-way hybrids) was produced crossing IL in replicates following a half-diallel scheme. Seeds were pooled by each pair to establish the bulk of 28 two-way lines. G_2_ (four-way hybrids) was produced crossing the 28 two-way in a half-diallel scheme. Pollen was pooled among three replicates for each two-way line and used to pollinate three ears. To maximize actual heterozygosity, only two-way hybrids with no parents in common were crossed to each other (210 individual crosses). Due to late flowering, four-way hybrids having HP301 x B96 as one of the two parents could not be produced, and the 2-way B73 x CML91 was introduced to the population to fill in the breeding gaps. The four-way hybrids were organized in 70 pools, each including balanced amounts of seeds of the three four-way hybrids carrying the same four alleles in different *cis* combinations (for example, ABxCD, ACxBD, ADxBC to constitute the pool ABCD). Pools were organized in 35 pairs in each of which all eight available alleles were represented (for example, ABCD & EFGH, CEFH & ABDG, and so on). G_3_ (eight-way hybrids) was produced crossing pools belonging to the same pair in 35 eight-way ABCDEFGH hybrids. Groups of three plants were pollinated at once using a pollen mix from five different plants. Twelve crosses were performed for each pair using individual plants either as male or female, not both. The first inbreeding generation was produced by selfing of eight-way hybrids in 2008, each subfamily (1 to 35) consisting of weighted pools of seeds from all the 12 corresponding four-way x four-way crosses. We sowed about 250 segregating seeds for each eight-way pool, and performed at least 50 selfings on randomly selected plants. Seeds from each ear that was selfed in 2008 were kept separate. The following season each field plot consisted of weighted seed pools from 10 such ears belonging to the same subfamily. A total of six such pools (60–80 seeds each) were sown as separate plots for each subfamily. Twenty random selfings per plot were performed, and single ears were hulled from selfed plants and numbered as #subfamily_#line. Ten seeds per ear (F_3_) were sown in Myanmar for the following winter generation of selfing. Five plants were left after thinning, two were selfed, and a random one was harvested. The same scheme was used to produce the F_5_ in summer 2010 (Italy) and the F_6_ in December 2010 (Myanmar). In summer 2011, RIL-F_6_ seed stocks were reproduced and expanded by sibbing, sowing 20 seeds for each line in separate plots, thinning to 10 plants and sibbing five plants. All produced seeds were harvested and bulked for each line. A total of 529 RIL-F_6_ were selected having the largest seed stock available while maintaining as much as possible a uniform representation of all 35 subfamilies, and as such underwent genotypic and phenotypic analyses.

### Field experiments and field data analysis

A sample of 529 RIL and replicates of each parental line (625 entries total) were field tested in 2012 at two locations in the Po valley, Rodigo (LAT 45.198927 LON 10.626078) and Caleppio (LAT 45.434038 LON 9.387104). Entries were field laid out as a 25 × 25 squared lattice design with two replicates per location [75], in single-row plots 4.40 m long and 0.80 m wide with a plant density of 6.25 plants m^2^. Plant density was kept low given the expected wide range of plant sizes so to minimize the biases due to uneven competition among plants of different size. Current field practices for maize were used, providing irrigations as needed to attain favorable growing conditions. Ears were hand harvested and shelled when uniform moisture was achieved. Data were collected on a single-plot basis for the following traits: (1) pollen shedding (PS), as the difference between PS and sowing date (assessed when 50% of plants had extruded anthers); (2) plant height (PH), measured on the flag leaf collar on three competitive plants per plot; (3) ear height (EH), measured on the node of the higher ear insertion; (4) grain yield per plant (GY), as the weight (adjusted to 15.5% moisture) in grams of the grains produced by each plot divided by the number of successful plants per plot. Variance components were estimated employing the following model:

1 $$ {y}_{ijkl}=\mu +{g}_i + {e}_j + g{e}_{ij}+{r}_{jk} + {b}_{kjl}+{\varepsilon}_{ijkl} $$

where *μ* is the overall mean, *g*_*i*_ the effect of the inbred line *i*, *e*_*j*_ is the effect of environment *j*, *ge*_*ij*_ the interaction between inbred line *i* within environment *j*, *r*_*jk*_ the effect of replication *k* within environment *j*, *b*_*kl*_ the effect of incomplete block *l* within replication *k*, and *ε*_*ijkl*_ the residual. All effects in Eq. (1) except *μ* were considered as random to estimate variance components and were computed by restricted maximum likelihood. A Wald test [76] was used to test significance of variances. In case of GY, the y_tr_ = y^0.5^ transformation was applied to obtain homoscedasticity of the residuals [77]. Heritabilities (*h*^*2*^) were calculated on an entry-mean basis, as the ratio of genotypic to phenotypic variance among RIL means [78]. Lines’ adjusted means were obtained as best linear unbiased estimates (BLUEs) considering μ, and *g*_*i*_ in Eq. (1) as fixed effects and the remaining effects as random. Computations were performed by using PROC MIXED in SAS (SAS Institute, Cary, NC, USA). Simple Pearson correlation coefficients (*r*) were calculated among all traits based on the adjusted means of the 529 lines.

### Genotyping and data processing

Seeds from the founder lines and the 529 RIL-F_6_ were sown in groups of five in petri dishes on moist paper (18h light /8h dark at 25 °C). Seedlings were collected and pooled from each petri dish. Genomic DNA was extracted from green tissues with SIGMA genelute plant genomic DNA miniprep kit (Sigma-Aldrich, St Louis, MO, USA). DNA was checked for quality and quantity on agarose gels and a NanoDrop ND-1000 spectrophotometer (Thermo Scientific, Wilmington, DE, USA). Samples having A_260_/A_230_ and A_260_/A_280_ above 1.5 were selected and assembled in microtiter plates randomizing subfamilies. MAGIC maize founder lines were genotyped in replica on the Illumina MaizeSNP50 BeadChip [20] at TraitGenetics (Gatersleben, Germany). Test F_1_ (B73XH99, B73xB96, B73xW153R, A632xB73, F7xB73, B73xMo17, and W153RxHP301) were also genotyped to asses correctness of markers’ segregation detection. Genotyping was performed in two experimental runs on 529 MM lines, organizing samples in randomized batches. Initial raw data processing and genotype calling was performed using GenomeStudio software 2011.1 (Illumina, Inc., San Diego, CA, USA). When one of the founder replicates contained an ‘N’ call at a marker, this was replaced with the allele call from the other replicate. For diversity analyses, markers were filtered to retain those polymorphic with a call rate over 80% in the full set of RIL. Samples were filtered for median emission intensity on the array as evaluated in R [79] with the package mclust [80]. Samples placing below the first percentile of the bivariate (x, y) density were removed from subsequent analyses (MM lines 15_73 and 19_36). The coordinates of the array oligo sequences were re-aligned to the reference genome sequence (B73 RefGenV3) using the software package BWA-MEM version 0.7.5a [81].

### Sequencing

Founder lines were sequenced to perform association mapping at QTL locations. At the time of sequencing, more than 10 years after the first cross, we were unable to germinate IL B96. For this reason this IL did not undergo full genome sequencing. Short reads produced on B96 with a GBS approach [82] were included in the dataset. The RefGenV3 sequence of B73 (reference genome [21]) was obtained from ensemble genomes [83]. Paired end reads from the genomic sequence of Mo17 were acquired from the Sequence Read Archive (SRA) [84]: SRR068224 (experiment SRX026937), SRR447948 (experiment SRX131285), SRR447949 (experiment SRX131286), SRR449556, SRR449557, and SRR449558 (experiment SRX132074). We sequenced the remaining five IL founders (A632, F7, H99, HP301, W153R) on the Illumina platform HiSeq2500 (Illumina, Inc., San Diego, CA, USA) with 101 cycles per read. DNA paired-end libraries were generated from genomic DNA, according to the standard Illumina paired-end sample preparation guide (Illumina Inc., San Diego, CA, USA), with slight modifications. Raw data was processed with the CASAVA 1.8.2 version of the Illumina pipeline. Raw sequences were quality trimmed and contaminant filtered using erne-filter version 1.2 (erne.sourceforge.net) and adapters were removed with cutadapt version 1.1 [85]. Short reads sequences were then mapped against the reference genome sequence (B73 RefGenV3) using the software package BWA-MEM version 0.7.5a [81] with the default settings. The aligner output was sorted and transformed to binary alignment/map (BAM) file with SAMtools version 0.1.18 [86]. PCR duplicates were removed with SAM tools and only uniquely aligned reads were retained. The variant discovery tool Unified Genotyper of the software package GATK version 2.8-1 [87] was used for SNP calling with heterozygosity parameter 0.01 [88, 89]. Raw SNPs were further filtered by quality (phred-scaled quality score >50) and by coverage of the SNP site (only the positions in the reference with a coverage ranging between 0.5 times and 1.5 times the modal values were considered). The filtered set of SNPs was used as a reference panel to impute missing position from B96. Impute2 [90] was used in subsequent 5 Mb windows to generate founders’ haplotypes. Genotypes were assigned on the base of higher probability, forcing null call to positions with less than 60% probability. Imputed genotypes were subsequently arranged in a VCF file filtering positions with an impute2 certainty metric lower than 0.9. Founder lines coverage data used for SV detection was generated using an internal pipeline, available upon request, which calculates how many times each position of the reference genome is covered by aligned reads. Read counts were normalized with the upper quartile method in edgeR [91].

### Expression

Transcriptome analysis was performed on proliferative tissue of eight founder lines (B73, H99, A632, CML91, F7, HP301, Mo17, W153). B96 did not undergo transcriptome analysis because of germination issues. Plants were grown in the growth chamber under controlled growth conditions (24 °C, 55% relative humidity, 170 μmol m^−2^ s^−1^ photosynthetically active radiation at plant level in a 16h/8h day/night cycle). We sampled for RNA extraction the most basal 0.5 cm of the fourth leaf during the steady state growth phase, that is, 3 days after the tip of the fourth leaf emerged from the pseudostem cylinder. At this stage the tissue is fully proliferative, as we determined by staining with 4′,6-diamidino-2-phenyindole (DAPI) as previously described [92]. Total RNA was extracted using TriZol. For B73 and H99, three biological repeats were performed, for the other founder lines two biological repeats, each consisting of a pool of four plants. Library preparation and sequencing was performed as described in [93]. Quality filtering was performed using FASTX-Toolkit ([94], version 0.0.13): reads were globally filtered in which for at least 75% of the reads the quality exceeds Q10 and 3’ trimming was performed to remove bases with a quality below Q20, ensuring a minimum length of 35 bp remaining. Re-pairing was performed using a custom perl script. Reads were mapped to the maize reference genome using GSNAP [95] allowing maximally five mismatches. The concordantly paired reads that mapped uniquely to the genome were used for quantification on the gene level with htseq-count from the HTSeq.py python package [96]. Data was normalized using trimmed mean of M-values (TMM), implemented in edgeR [91]. K-means clustering of the QTL coefficients to test for differential expression was performed using R/fpc [97]. The number of clusters in which founder coefficients could be grouped in QTL confidence intervals was determined according to the average silhouette width. We grouped the most extreme founder effects and compared them to the rest, determining differential expression between groups using a generalized linear model in edgeR [91]. edgeR employs a negative binomial distribution to fit the count data, and proved to be superior to correlative methods in comparing contrasting founder effects. For each gene within the confidence interval and within the ± 1 Mb around it, we compared differential expression testing separately the high effect founders versus the rest and the low effect founders versus the rest. We applied a multiple testing correction with R/qvalue [98], setting a FDR threshold of 0.05.

### Diversity analyses

The filtered set of allele calls was used to survey the diversity comprised in the RIL population. The Bioconductor package snpStats [99] was used to compute the basic diversity indexes and to compare minor allele frequency (MAF) between founders and RIL. Only completely homozygous SNP successfully genotyped in all eight founders plus CML91 were considered to compute MAF. The same subset of SNP was extracted from the MM lines dataset and used for MAF comparison. The package R/SNPRelate [100] was used to evaluate the structuration of the RIL population by computing a principal component analysis (PCA) extracting the first 100 PC. The set of mapped SNPs was used to compare the PC assortment of MM lines when considering SNPs falling in pericentromeric and telomeric regions. We obtained physical location and span of centromeres from the maize genome assembly, and SNPs were deemed centromeric when falling ± 1 Mbp of the pericentromeric range. The telomeric set of SNPs was obtained selecting those falling in the same physical span in one of the telomeric arms for each chromosome. Telomeric and pericentromeric SNP were used to separately compute a PCA. The package R/adegenet 1.3 was used to perform a discriminant analysis of principal components (DAPC) [101]. The function snpgdsLDpruning in R/SNPRelate was used to generate a set of SNP in approximate linkage equilibrium (threshold 0.4 *r*^*2*^) by recursively dropping SNP in high linkage within 500 Kb windows. The subset of SNP was used to build a Neighbor Joining (NJ) tree in R/ape [102] using parsimony substitution models.

### Linkage disequilibrium

Linkage disequilibrium analysis was performed on mapped array SNPs with the package R/LDheatmap [103]. The *r*^*2*^ measure was preferred over *D*’ as takes in account allele frequencies and weights co-inheriting by MAF at each locus, more appropriate when not all loci are informative as in a MpCD. The evolution of LD as a function of physical distances was evaluated considering pairwise LD measures within 10 Mb on each chromosome separately and calculating average *r*^*2*^ in 100 Kb windows. The LD halving distance was calculated for each chromosome independently. To visualize local LD decay, we considered each marker separately, averaging for each the pairwise *r*^*2*^ with all surrounding markers within ±1 Mb. This value was chosen as 2 Mb is the intermediate halving distance of LD according to LD decay analysis. Individual markers’ *r*^*2*^ were averaged for each chromosome in sliding windows considering 100 markers at once. The same set of parameters was used to compute and plot the 25^th^ and 75^th^ marker-centered percentiles of the LD values distribution so to observe regions of potential disagreement between the two distributions. Different window sizes were also tested, as the interval in which LD is averaged affects the summary statistic generated. The size chosen proved to be the better in depicting local peaks of consistently higher LD.

### RIL genome reconstruction

We reconstructed the genomes of the MM lines using a hidden Markov model (HMM) that produces a probabilistic reconstruction of each MM genome. We modified the allele call based HMM in an existing R package (DOQTL) [12] to reconstruct the MM genomes. The HMM contains eight or nine homozygous genotype states, depending on the number of founders that contributed to each line. The HMM requires three sets of input data: (1) prior probabilities for each genotype state; (2) the probability of observing each allele given the genotype state at each marker (emission probabilities); and (3) the probability of observing a recombination between markers (transition probabilities). The prior probabilities were set to one divided by the number of founders that contributed to each line. The initial emission probabilities were calculated based on the allele frequencies in the founder lines. At markers where the founders were heterozygous, we distributed the probability between the two homozygous alleles. In order to allow for uncertainty in the emission probabilities, we added 0.01 to states with low probability and subtracted an amount from the other states such that the probabilities for one state summed to one. The transition probabilities were obtained from the two point recombination probabilities for eight-way crosses produced by selfing [23] as:

2 $$ \frac{r\left(4-r\right)}{1+2r} $$

where *r* is the recombination fraction between two markers. We calculated *r*/*100* as the cM distance divided by 10^6^ as a tuning parameter. Once the initial HMM was composed, we updated the emission probabilities using the Expectation-Maximization (EM) algorithm [104, 105]. In the E-step, we calculated the MM genotype probabilities using the HMM. In the M-step, we updated the emission probabilities based on the MM genotype probabilities. We ran the model until the log-likelihood of the HMM differed by less than 1/1,000th of the initial log-likelihood. The genotyping HMM produces estimates of the probability that each founder line contributed to each MM line at each marker. The expected founder contribution to the MM population was calculated from the doses contributed by each parental genome accordingly to the breeding design. The expected number of recombinations was calculated as follows: assuming a genetic map 19.96 M long, we counted 19.96 recombinations for each of the three generations of intermating (G_1_, G_2_, and the first selfing in G_3_), adding additional 19.96 recombinations throughout the inbreeding generations.

### QTL mapping

The MM lines may have complex genetic relationships and this must be accounted for in the mapping model [70]. We included an adjustment for the kinship between the MM lines. We calculated the kinship matrix based on the inner product of the genotype probabilities between each pair of lines. We performed two types of genetic mapping: linkage mapping and association mapping. We performed linkage mapping by regressing phenotypes on the genotype probabilities produced by the HMM. Due to the low allele frequency of CML91 at each marker, the mapping model became unstable and produced large coefficient estimates for CML91. For each sample with a non-zero CML91 contribution, we removed the CML91 values and normalized the remaining founder proportions to sum to 1 and used these in mapping. We used the R package QTLRel [106] to fit this model because it includes an adjustment for the kinship between lines. These relationships will introduce correlation in the model residuals that may inflate the type I error [70]. The linkage mapping model is:

3 $$ {y}_i={\displaystyle \sum_{s=1}^8{p}_{ij}(s){\beta}_s}+{\gamma}_i+{\varepsilon}_i $$

where *y*_*i*_ is the phenotype for line *i. p*_*ij*_(*s*) is the genotype probability produced by the HMM, *β*_*s*_ is the effect of founder *s*, *γ*_*i*_ is a random effect with covariance *σ*_g_^2^K and *ε*_*i*_ is a random effect with covariance *σ*_e_^2^I [13]. The LOCO (Leave One Chromosome Out) method was applied to kinship calculation. In this method each chromosome scan is conducted considering kinship calculated on all chromosomes but the current one, limiting the correction applied locally to QTL scan. Significance thresholds to call a QTL were calculated by 1,000 permutations of each phenotypic trait. The 99^th^ percentile of the permuted LOD distribution was chosen as a high significance threshold for each trait. The 37^th^ percentile was reported as a suggestive significance threshold for all traits [28]. QTL analysis was repeated for each trait by including the largest QTL as a covariate. Confidence intervals are flanked by the closest markers at −2 LOD from the highest peak. Different peaks within the same confidence interval were reported individually when separated by at least 50 markers with a LOD score below the suggestive threshold. We performed association mapping by imputing the founder line SNP onto the MM genomes [12]. The regression equation at each marker is:

4 $$ {y}_i={g}_{ij}{\beta}_g+{\gamma}_i+{\varepsilon}_i $$

where *g*_*ij*_ is the genotype of line *i*, *β*_*g*_ is the additive effect of each allele and the remaining terms are as in Eq. (1). We used the genotypes resulting from imputation to perform association mapping on a single chromosome at a time. The 90^th^ percentile of 500 permuted LOD distributions was used as the significance threshold. Best orthologs of MM candidate genes were identified by Plaza 3.0 integrative orthology method [29]. When no best orthologs could be identified, sequence homology was surveyed with Plaza 3.0 and Gramene [107]. LD was calculated in the QTL confidence interval using imputed SNP. Imputation probabilities based on haplotype reconstruction were rounded to either 0 or 1 and used as binary SNPs in R/LDheatmap [103] to compute *r*^*2*^. Subsequently, for each SNP *r*^*2*^ was averaged in a window of 100 Kb on each side, and *r*^*2*^ evolution was calculated applying a rolling window of size based upon the average number of markers present in a 200 Kb interval.

### Phenotype simulation

We simulated QTL in a manner similar to [25]. We simulated QTL using scenarios with four minor allele frequencies (MAF = 1, 2, 3, or 4 founders), five sample sizes (n = 100, 200, 300, 400, or 500 lines) and two additive heritabilities (*h*^*2*^ = 0.4 or 0.7). We define a scenario as the unique combination of MAF, n and *h*^*2*^. We performed 100 simulations for each scenario (that is, 100 simulations for MAF = 1, sample size = 100, and h = 0.4). For each, we randomly selected a subset of the 529 lines. We generated 20 effect sizes with a geometric distribution such that QTL *i* had an effect size of 0.9^*i*^. We randomly selected 20 markers, two per chromosome, as simulated QTL locations. At each marker, we randomly selected a different set of MAF founders, *F*, to contribute the minor allele (that is, for MAF = 2, at QTL 1, founders A and C contributed the effect, at QTL 2, founders B and F contributed the effect, and so on). For each line, we obtained the founder haplotype probabilities and condensed them down to a genotype indicating the allelic contribution of the founders contributing the QTL effect:

5 $$ {f}_{ij}=2\left[{\displaystyle {\sum}_{s=1}^9I\left(s\in F\right){P}_{ij}(s)}\right]-1 $$

where *f*_*ij*_ is the founder contribution at marker *i* of individual *j*, *I*(*s* ∈ *F*) is an indicator that is 1 if founder *s* is among the founders, *F*, contributing the QTL effect at QTL *i*, and *P*_*ij*_ (*s*) is the haplotype probability for founder *s* at marker *i* for individual *j*. The genetic effect of each line was the sum of all 20 genetic effects. We scaled the variance of the genetic effect equal to 1 and generated Gaussian noise with mean = 0 and variance = 1. We summed the genetic effect and the random noise, scaling the genetic effect to contribute 40% (*h*^*2*^ = 0.4) or 70% (*h*^*2*^ = 0.7) of the total variance. The script for this procedure is available upon request.

### Simulated phenotype mapping

We mapped the simulated QTL in a manner similar to [25]. We performed single marker mapping at each of the markers using Eq. (1). We selected the marker with the highest LOD score and performed a likelihood ratio test (LRT), asking if this marker should be added to the model when compared with the reduced model without the current marker. The marker was added if the LRT χ^2^*P* value was ≤0.01. We scanned the genome again, including the new marker in the model, and markers were added until no marker could be added with a *P* ≤0.01. The markers locations included in the model were compared with the simulated QTL locations and a QTL was considered detected if it fell within +/− 5 Mb of the simulated QTL location. Power was calculated as the proportion of times a simulated QTL was detected. The false discovery rate (FDR) was calculated as the proportion of non-simulated QTL that were mapped over the total number of QTL mapped.

### Data availability

Full sequences of the founder lines A632, F7, H99, HP301, and W153R are available at the Sequence Read Archive (http://www.ncbi.nlm.nih.gov/sra) under BioProject PRJNA272385. Transcriptomics data are available at ArrayExpress (https://www.ebi.ac.uk/arrayexpress/) under accession number E-MTAB-3173. Genotypic data of the MM founders and MM lines are available at Figshare, DOI: http://dx.doi.org/10.6084/m9.figshare.1437453 and DOI: http://dx.doi.org/10.6084/m9.figshare.1437449, respectively. Imputed SNPs for the MM founders are also available at Figshare, DOI; http://dx.doi.org/10.6084/m9.figshare.1425350. MAGIC maize lines are stored at Scuola Superiore Sant’Anna (IT) and are available to researchers from public institutions free of charge. MM seeds and any additional genotyping data further produced should be requested sending an e-mail to: magic.maize.inbox@gmail.com.

## Additional files

Additional file 1: Table S1. Details of the MAGIC maize founder lines. For each line, developer, breeding group, and pedigree are given. Web links to further information and seed availability are provided. (XLSX 9 kb) Additional file 2: Table S2. MM population breeding design, organized in 35 subfamilies. In blue and red, single and double introductions of the backup founder (CML91). (XLSX 23 kb) Additional file 3: Figure S1. Distribution of heterozygous markers in the MM population genomes. Panel **a** shows average heterozygosity in telomeric regions (white bars) and pericentromeric regions (black bars). Pericentromeric regions are defined as 20 cM windows around centromeric positions. Panel **b** shows heterozygosity averaged over 1 Mb bins across the MM lines genomes (proportion of heterozygosity increasing from white to red, as reported in the right bar). There are few positional enrichments of heterozygosity, the most marked in Chr 8 pericentromeric region, as evident from panel **a**. (TIFF 133 kb) Additional file 4: Figure S2. Chromosome-wise distribution of polymorphic markers (as fraction of markers polymorphic per 1 Mb bin, gray line), marker density (as number of markers standardized per chromosome, yellow shading), and heterozygosity (as average heterozygosity in 1 Mb bins, red line). Note the two different scales on the y axes. Centromere positions are marked with black triangles. The observed heterozygosity is not related to polymorphism distribution. Marked enrichments for heterozygous loci are present on Chr 3, Chr 8, and Chr 10, as apparent from Additional file 3: Figure S1. (PDF 709 kb) Additional file 5: Figure S3. Unrooted phylogeny of the MM population, magnified from Fig. 3a. Founders’ placement is highlighted with the corresponding colors. Note that genetic distances between MM lines are evenly distributed throughout. (PDF 20148 kb) Additional file 6: Figure S4. Structure in the MM population. A principal components (PC) analysis on MM founders genotypes is shown in panel **a**. PC one to five are shown, each square representing one founder with colors according to those given in Fig. 1. Two replicas for each founder are shown. As expected, PC loadings (and structure) for founders are higher than those of RIL. Panel **b** shows PC one to five for the MM lines. Breeding subfamilies are depicted in different colors. PC 1 provides a slight separation in two clusters, but the highest PC loading is of only 1.7%. The distribution of the PC loadings for PC 1–100 in the MM lines shows a smooth decrease throughout in panel **c**. Panel **d** reports the outcome of a discriminant analysis of principal components (DAPC) over the MM lines. The lowest BIC values are assigned to one-two clusters, confirming the absence of structuration in the MM population. (TIFF 3269 kb) Additional file 7: Figure S5. Detail of the local LD decay distribution along each chromosome. The 25^th^ percentile (blue line) and 75^th^ percentile (red line) of the LD distribution within ±1 Mb each marker is shown. Centromere positions are marked with black triangles. The r2 quantiles for each marker-based window are averaged in sliding windows of 100 markers in size. Some deviations between the general shape of the two distributions are evident, notably in Chr 2 and Chr 6. (PDF 12383 kb) Additional file 8: Table S3. The genetic map of the MM population. (XLSX 2159 kb) Additional file 9: Figure S6. Genomic composition of the MM population. Panel **a** shows the distribution of the number of recombination events in MM RIL. The observed mean value (80.9) is close to the expectancy (79.8; see text). In panel **b** colored bars refer to each founder contribution to the MM population, as calculated from MM genome reconstruction. On top of the bars, the observed contribution in percentage. The red line refers to one-eighth, or 12.5%. IBS regions also influence parental contribution estimation. Panel **c** shows mean genomic similarity of founder lines per chromosome (increasing similarity from red to white as reported by the scale on the right). (PDF 2927 kb) Additional file 10: Table S4. Quantitative data for MM founders’ sequencing and expression. For sequencing, raw reads count and mean and modal coverage (in folds) are given. For expression data, raw reads numbers are given for each biological replica (B1, B2, B3). (XLSX 9 kb) Additional file 11: Table S5. Summary of power simulation results with varying sample sizes (100, 200, 300, 400, 500). For each simulated QTL, the effect and the variance explained are given. The power to detect each QTL is averaged on 400 independent runs (100 for each of MAF 0.125, 0.25, 0.375, and 0.5). Results are given for *h* ^*2*^= 0.4 and 0.7. (XLSX 17 kb) Additional file 12: Figure S7. Average power of the MAGIC maize population as a function of the number of lines analyzed. Plot design and simulation approach refer to power simulations run on the NAM population. Note the number of lines on the x axis, one order of magnitude lower than the NAM. Panel A reports the case with 20 QTL simulated with heritability 0.4, panel B with heritability 0.7. (TIFF 1059 kb) Additional file 13: Table S6. Phenotypic estimated values of the eight founders and 529 MM lines analyzed for days to pollen shed (PS), plant height (PH), ear height (EH), and transformed grain yield (GYrad). (XLSX 36 kb) Additional file 14: Figure S8. Distribution of phenotypic estimated values across the MM lines. Frequency classes of MM lines are chosen on the basis of the standard error of the mean for each traits. Grain yield is y_tr_ = y^0.5^ transformed to obtain homoscedasticity of the residuals. Plant height and ear height are in the range of 76.8–252.1 cm and 32.3–164.91 cm, respectively. Days to pollen shed variation spans 16 days. (TIFF 578 kb) Additional file 15: Table S7. Mean, range, genotypic variance (*σ* ^*2*^_*g*_), genotype × environment interaction variance (*σ* ^*2*^_*ge*_), residual (*σ* ^*2*^_ɛ_), and heritability (*h* ^*2*^) of the MM lines for the investigated traits. (XLSX 9 kb) Additional file 16: Figure S9. QTL scan for grain yield. In panel **a**, the full model scan. On the x axis, the physical position from Chr 1 to 10. On the y axis, the LOD score. Red and green thresholds represent strong (*P* <0.01) and suggestive (*P* <0.63) thresholds by 1,000 permutations, respectively. In panel **b**, the scan for the same trait including the highest QTL in panel a as a covariate. (PDF 1589 kb) Additional file 17: Figure S10. QTL scan for ear height. In panel a, the full model scan. On the x axis, the physical position from Chr 1 to 10. On the y axis, the LOD score. Red and green thresholds represent strong (*P* <0.01) and suggestive (*P* <0.63) thresholds by 1,000 permutations, respectively. In panel **b**, the scan for the same trait including the highest QTL in panel a as a covariate. (PDF 2003 kb) Additional file 18: Figure S11. QTL scan for plant height. In panel **a**, the full model scan. On the x axis, the physical position from Chr 1 to 10. On the y axis, the LOD score. Red and green thresholds represent strong (*P* <0.01) and suggestive (*P* <0.63) thresholds by 1,000 permutations, respectively. In panel **b**, the scan for the same trait including the highest QTL in panel a as a covariate. (PDF 1607 kb) Additional file 19: Figure S12. QTL scan for days to pollen shed. In panel **a**, the full model scan. On the x axis, the physical position from Chr 1 to 10. On the y axis, the LOD score. Red and green thresholds represent strong (*P* <0.01) and suggestive (*P* <0.63) thresholds by 1,000 permutations, respectively. In panel **b**, the scan for the same trait including the highest QTL in panel a as a covariate. (PDF 1618 kb) Additional file 20: Table S8. Suggestive loci (*P* <0.63) for days to pollen shed (PS), ear height (EH), plant height (PH), and grain yield (GY). Each row reports the phenotype (Trait), the covariates (Cov) eventually used in the QTL scan, the name of the marker (Marker) and chromosome (Chr) of the current association, the genomic position (in bp) of the interval determined by a 2 LOD drop from the highest association (Top), and the left (Left bound) and right (Right bound) bounds of this interval. The percent of variance explained (Variance) and the maximum LOD score are also shown. To avoid duplication, when a peak position was unchanged after scanning with a covariate, the latter scan is the only reported. QTL are listed individually if separated by at least 50 markers with LOD score below the suggestive threshold. (XLSX 14 kb) Additional file 21: Table S9. Genes whose expression in the founder lines matches founder effects (FDR <0.05) in ± 1 Mb QTL intervals for GY. For each gene, chromosome (chr), test (high (H) or low (L)), *P* value (pval), q value (qval), and start and stop positions are given. (XLSX 11 kb) Additional file 22: Table S10. Genes whose expression in the founder lines matches founder effects (FDR <0.05) in ± 1 Mb QTL intervals for PS. For each gene, chromosome (chr), test (high (H) or low (L)), *P* value (pval), q value (qval), and start and stop positions are given. (XLSX 17 kb) Additional file 23: Figure S13. Linkage disequilibrium within the flowering time QTL confidence interval. Panel **a** shows the heat map considering all imputed SNPs in the region. From white to red, increasing LD. Along the diagonal, SNP marker index, not proportional to physical position (noted on edges). *ZCN8* position is marked with an asterisk. The haplotype of highest significance is framed in blue. Panel **b** shows mean LD in a sliding window. On the x axis, physical position on the genome. The red box marks the haplotype of highest significance according to the association approach. LD is generally low in the QTL region, but a few distinctive peaks are visible: the most central to the QTL interval is short upstream *ZCN8* (position marked with *), which lacks imputed SNPs. (PDF 2509 kb)

## Abbreviations

CC: Collaborative cross
Chr: Chromosome
cM: CentiMorgan
cM: centiMorgan
DO: Diversity outbred
EH: Ear height
FDR: False discovery rate
GY: Grain yield
HMM: Hidden Markov model
IBD: Identity by descent
IBM: Intermated B73 x Mo17
IBS: Identity by state
LD: Linkage disequilibrium
LOD: Logarithm of odds
MAF: Minor allele frequency
MAGIC: Multi-parent advanced intercrosses
Mb: Megabases
MM: MAGIC maize
MpCD: Multi-parent cross designs
NAM: Nested association mapping
NIH-HS: Heterogeneous stock
NJ: Neighbor joining
PC: Principal component
PCA: Principal component analysis
PH: Plant height
PS: Days to pollen shed
QTL: Quantitative trait loci
RIL: Recombinant inbred lines
RIX: Recombinant inbred intercrosses
SNP: Single nucleotide polymorphism
SV: Structural variation
