# Supplementary figures and images for: Genetic properties of the MAGIC maize population: a new platform for high definition QTL mapping in Zea mays
Source: Genome Biol. 2015 Sep 11;16(1):167. doi: 10.1186/s13059-015-0716-z (PMC4566846; doi:10.1186/s13059-015-0716-z)

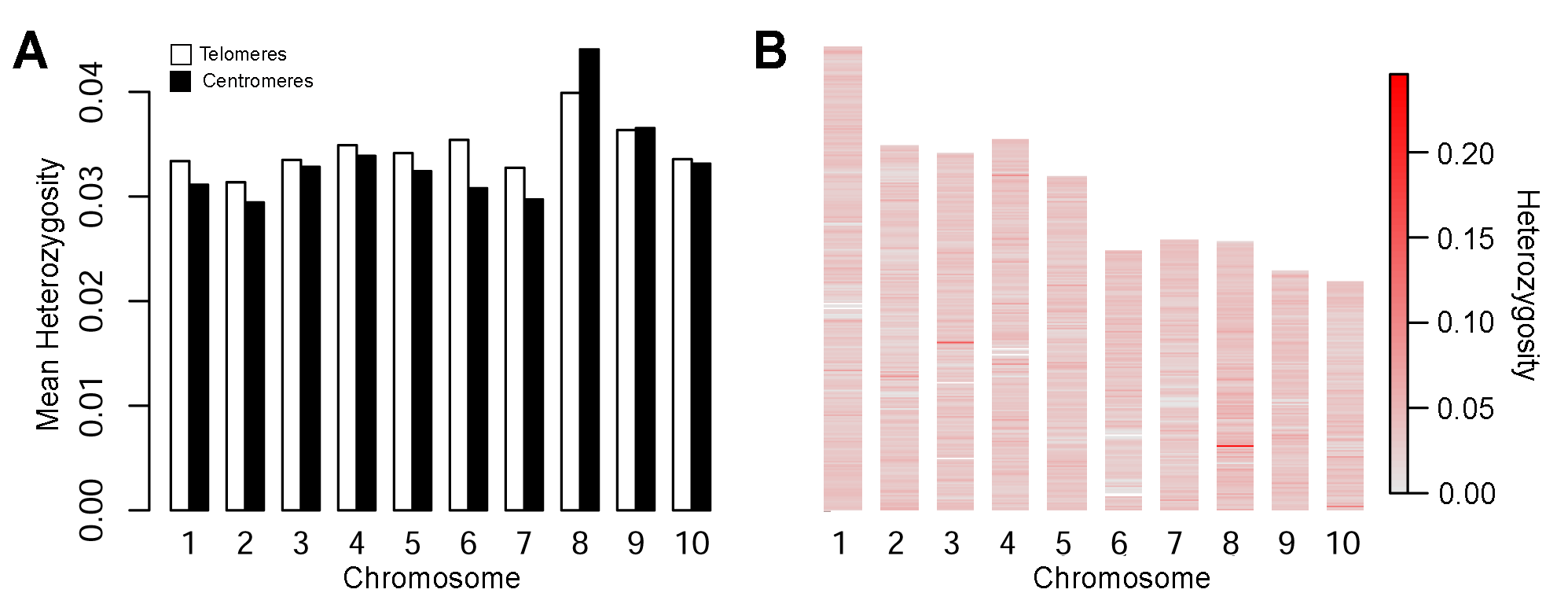

Supplement: Additional file 3: Figure S1. — Distribution of heterozygous markers in the MM population genomes. Panel a shows average heterozygosity in telomeric regions (white bars) and pericentromeric regions (black bars). Pericentromeric regions are defined as 20 cM windows around centromeric positions. Panel b shows heterozygosity averaged over 1 Mb bins across the MM lines genomes (proportion of heterozygosity increasing from white to red, as reported in the right bar). There are few positional enrichments of heterozygosity, the most marked in Chr 8 pericentromeric region, as evident from panel a. (TIFF 133 kb) [file 13059_2015_716_MOESM3_ESM.tif]

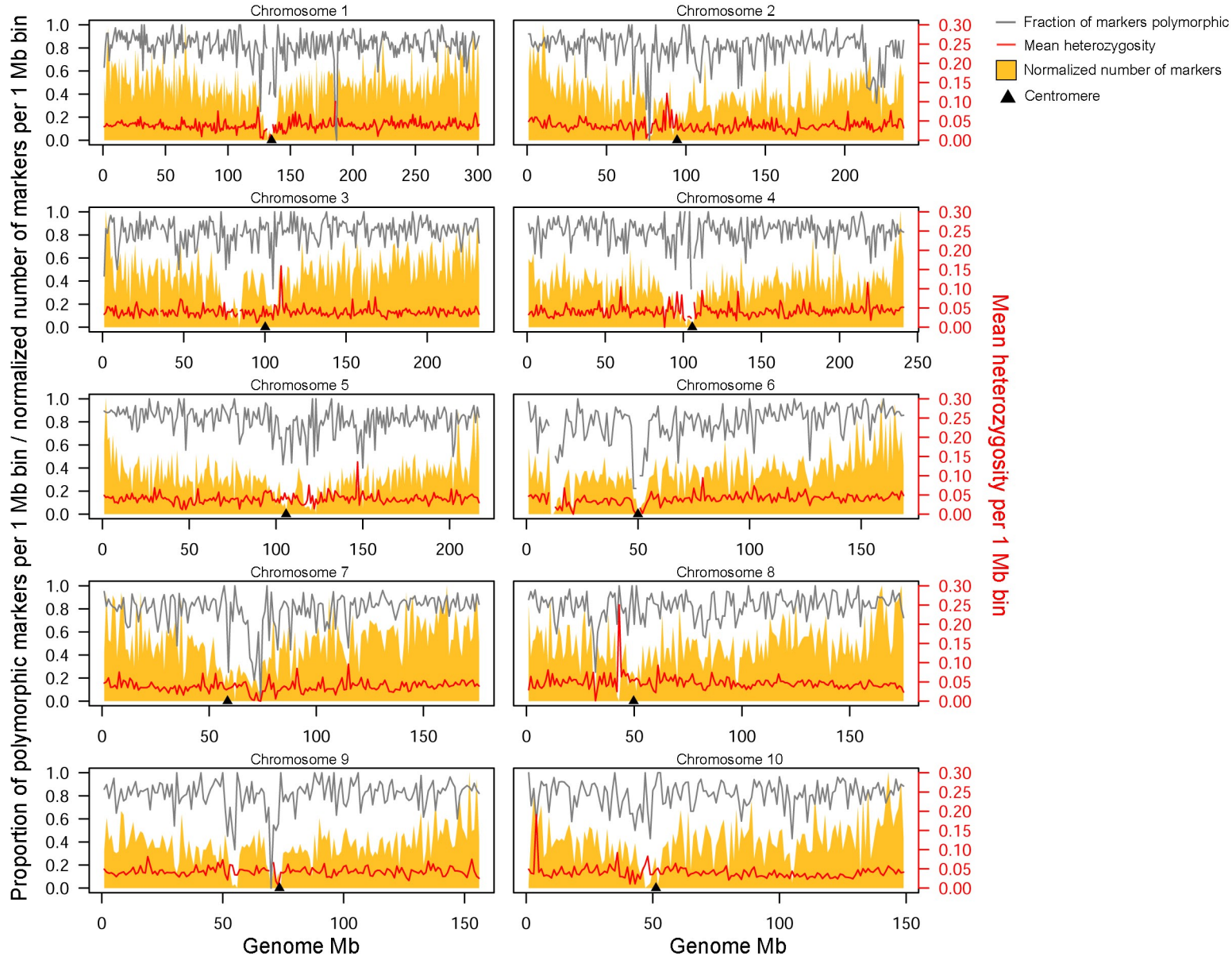

Supplement: Additional file 4: Figure S2. — Chromosome-wise distribution of polymorphic markers (as fraction of markers polymorphic per 1 Mb bin, gray line), marker density (as number of markers standardized per chromosome, yellow shading), and heterozygosity (as average heterozygosity in 1 Mb bins, red line). Note the two different scales on the y axes. Centromere positions are marked with black triangles. The observed heterozygosity is not related to polymorphism distribution. Marked enrichments for heterozygous loci are present on Chr 3, Chr 8, and Chr 10, as apparent from Additional file 3: Figure S1. (PDF 709 kb) [file 13059_2015_716_MOESM4_ESM.pdf]

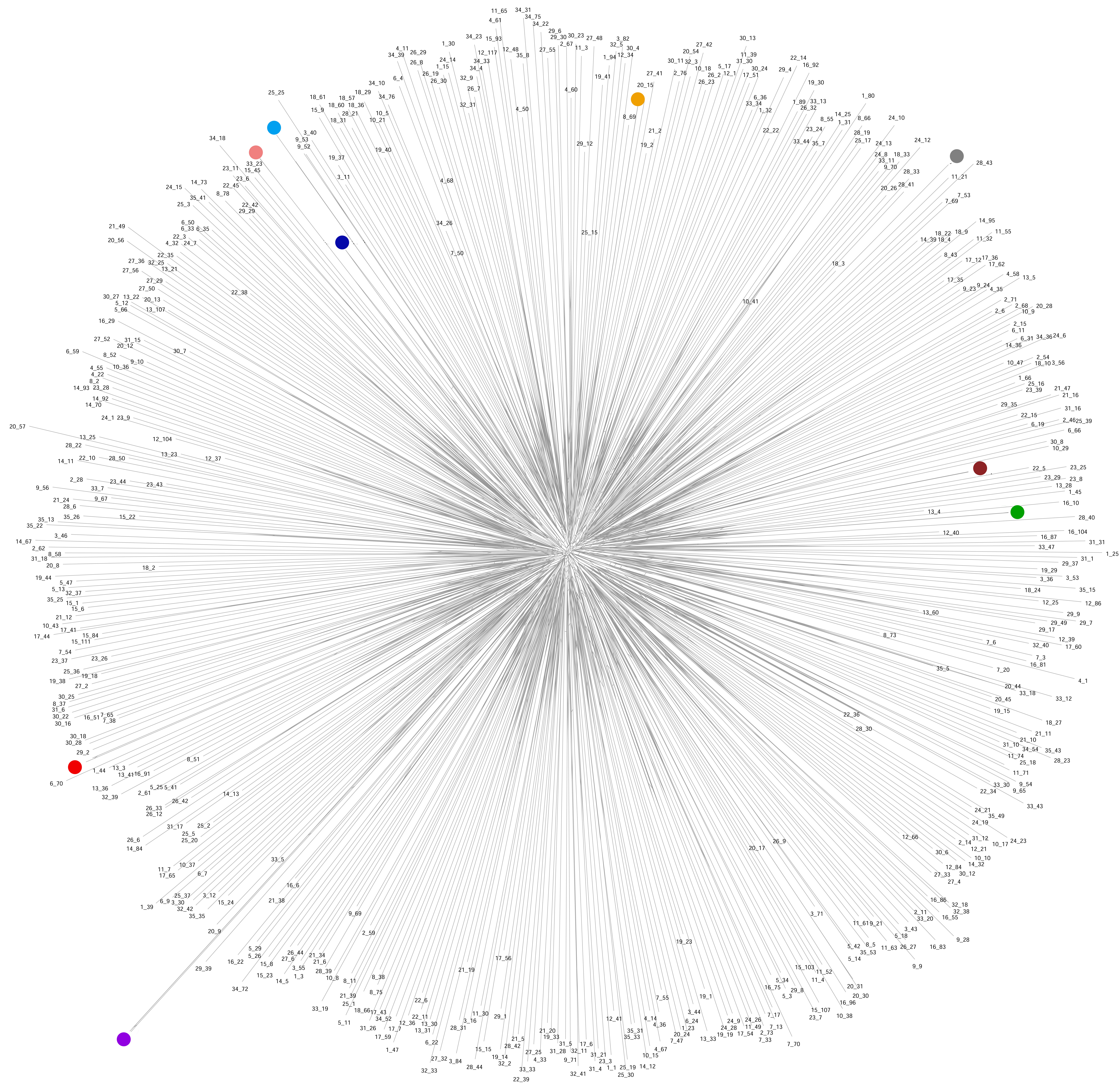

Supplement: Additional file 5: Figure S3. — Unrooted phylogeny of the MM population, magnified from Fig. 3a. Founders’ placement is highlighted with the corresponding colors. Note that genetic distances between MM lines are evenly distributed throughout. (PDF 20148 kb) [file 13059_2015_716_MOESM5_ESM.pdf]

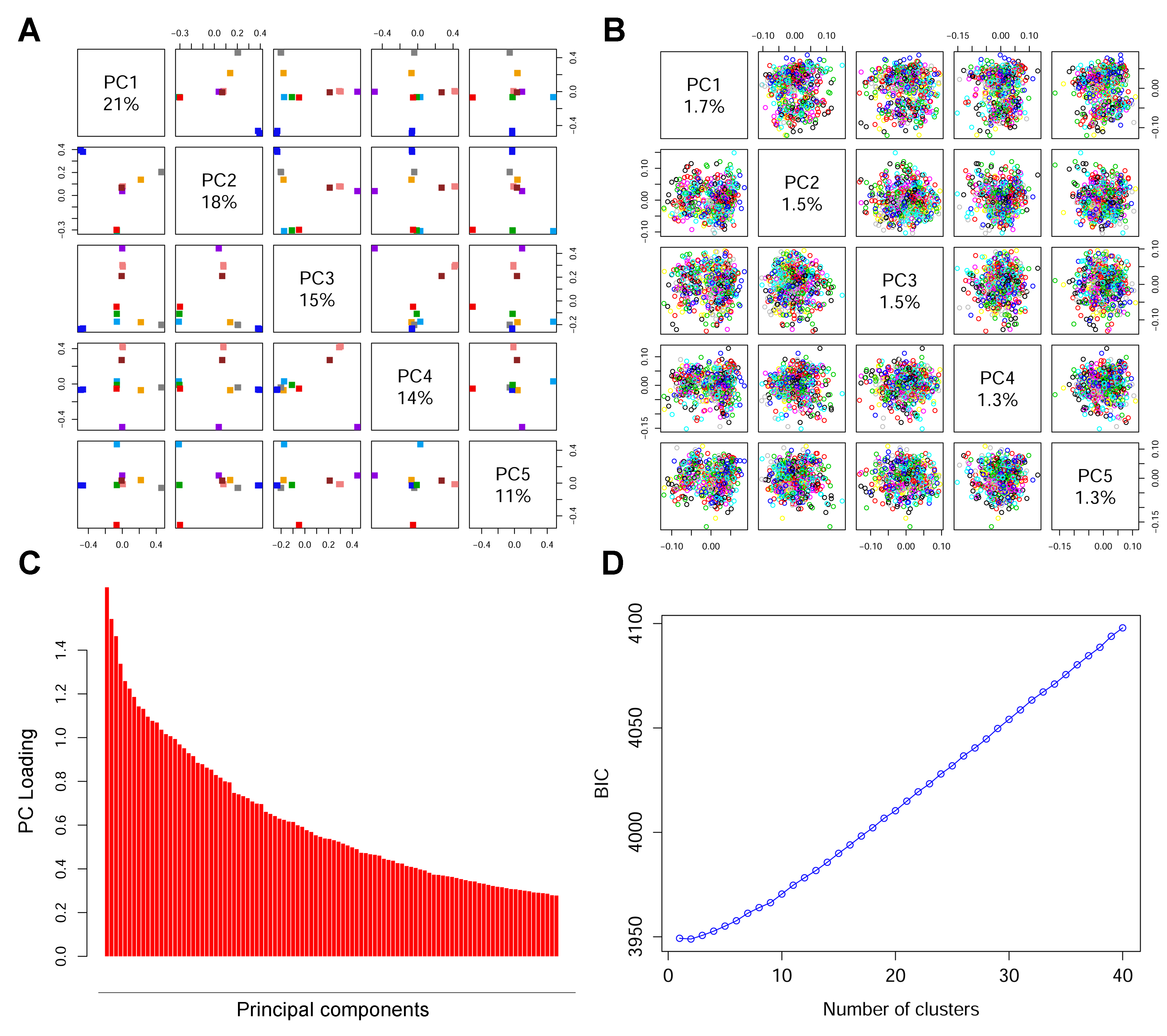

Supplement: Additional file 6: Figure S4. — Structure in the MM population. A principal components (PC) analysis on MM founders genotypes is shown in panel a. PC one to five are shown, each square representing one founder with colors according to those given in Fig. 1. Two replicas for each founder are shown. As expected, PC loadings (and structure) for founders are higher than those of RIL. Panel b shows PC one to five for the MM lines. Breeding subfamilies are depicted in different colors. PC 1 provides a slight separation in two clusters, but the highest PC loading is of only 1.7%. The distribution of the PC loadings for PC 1–100 in the MM lines shows a smooth decrease throughout in panel c. Panel d reports the outcome of a discriminant analysis of principal components (DAPC) over the MM lines. The lowest BIC values are assigned to one-two clusters, confirming the absence of structuration in the MM population. (TIFF 3269 kb) [file 13059_2015_716_MOESM6_ESM.tif]

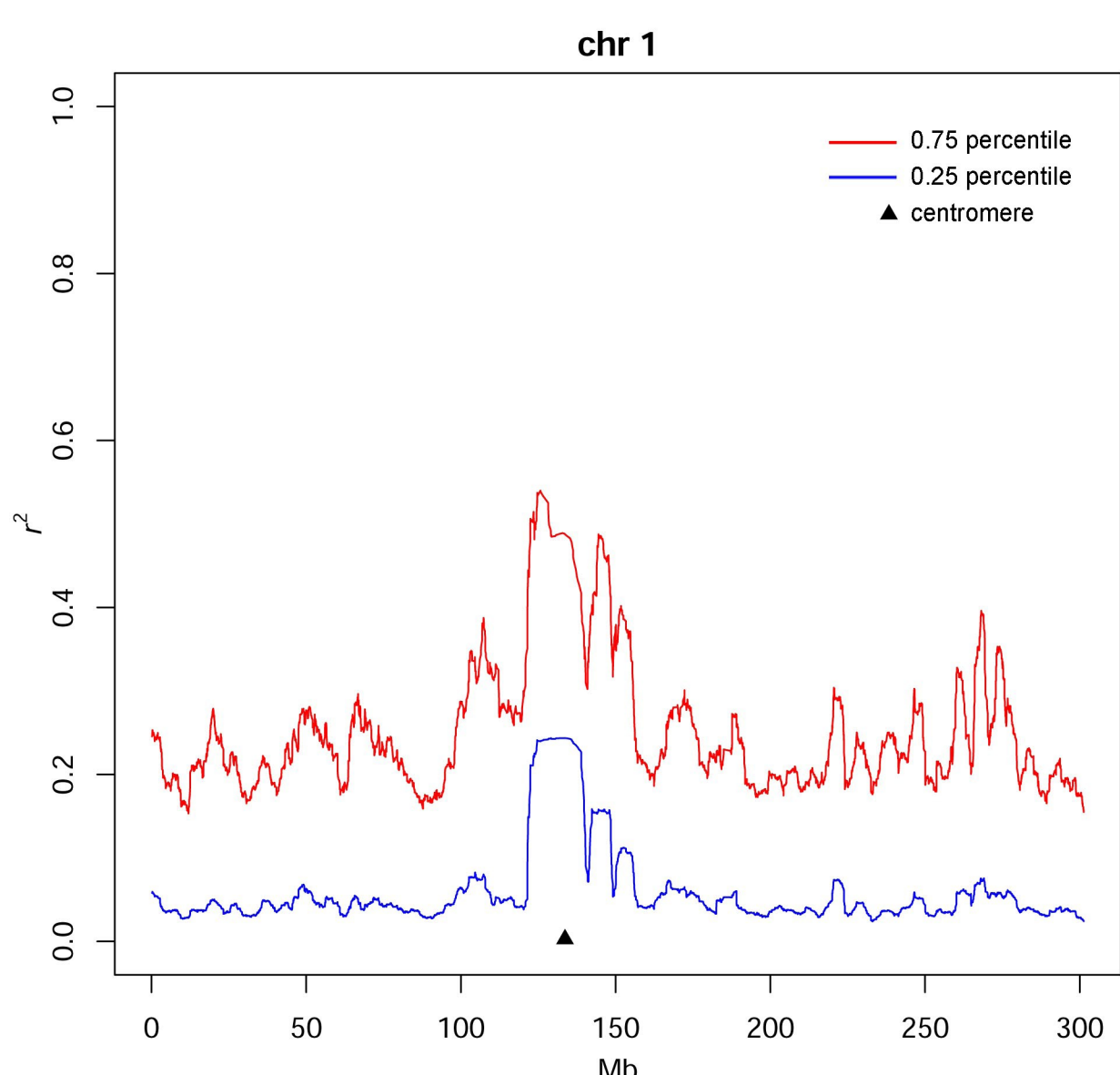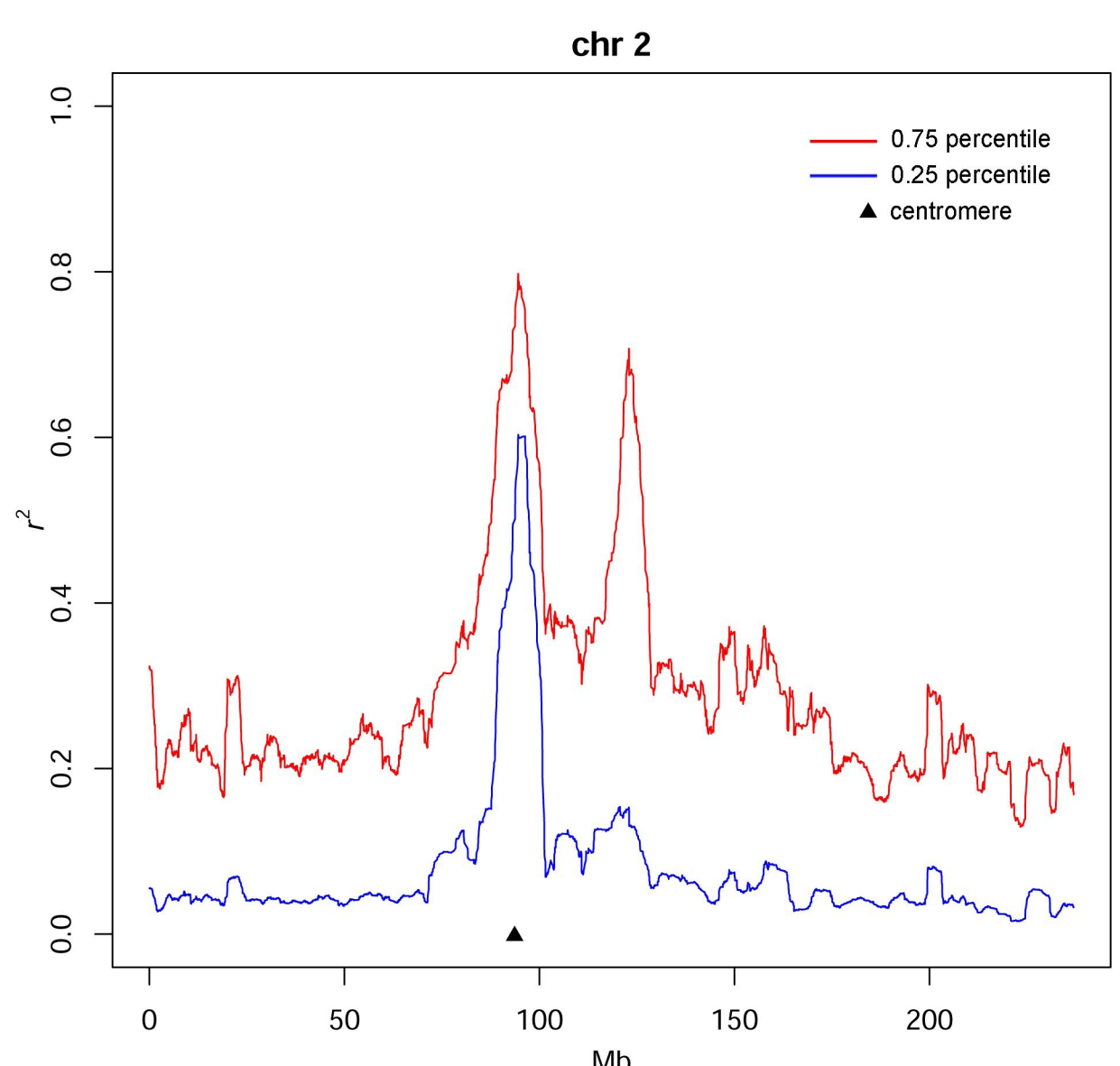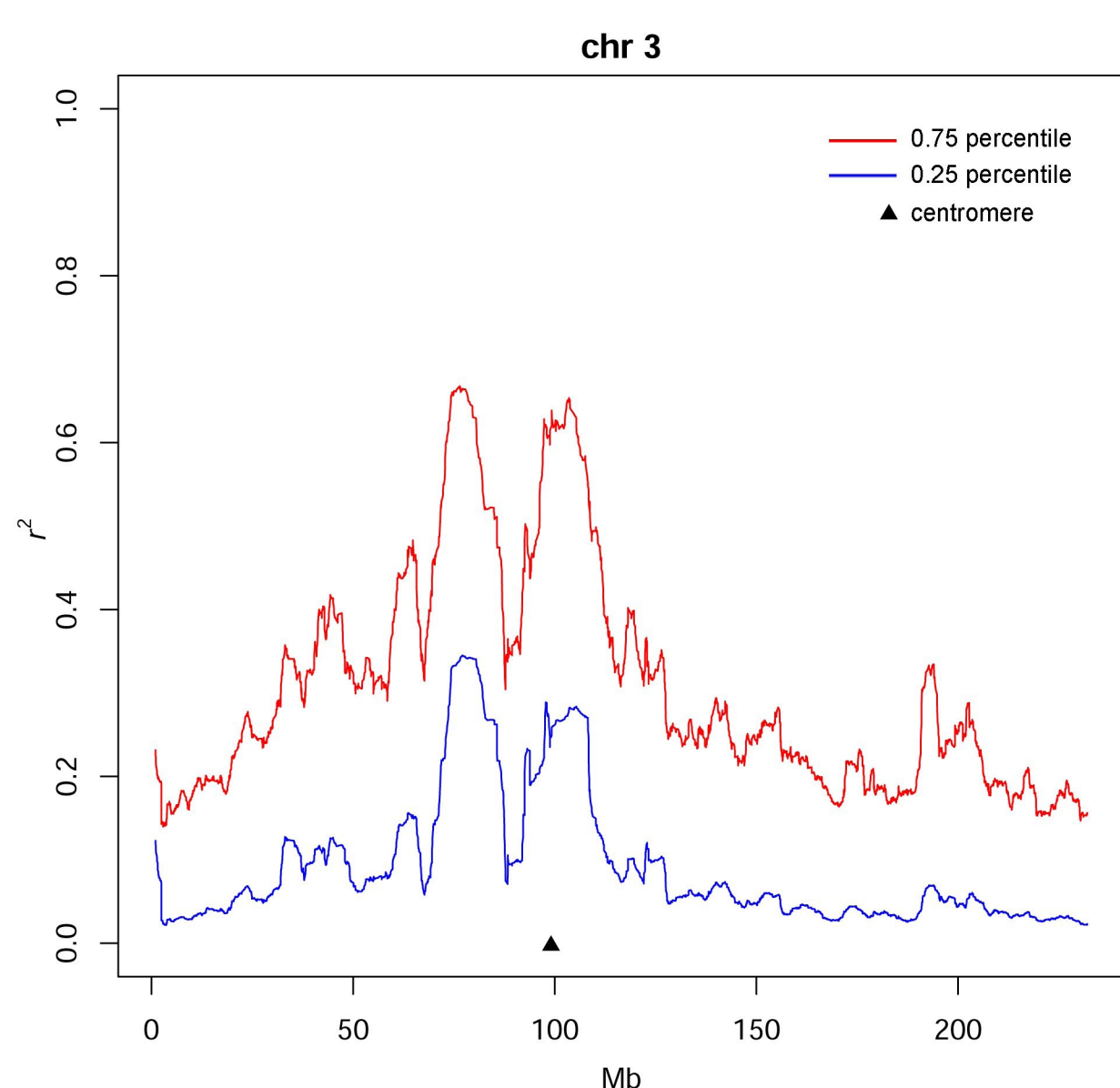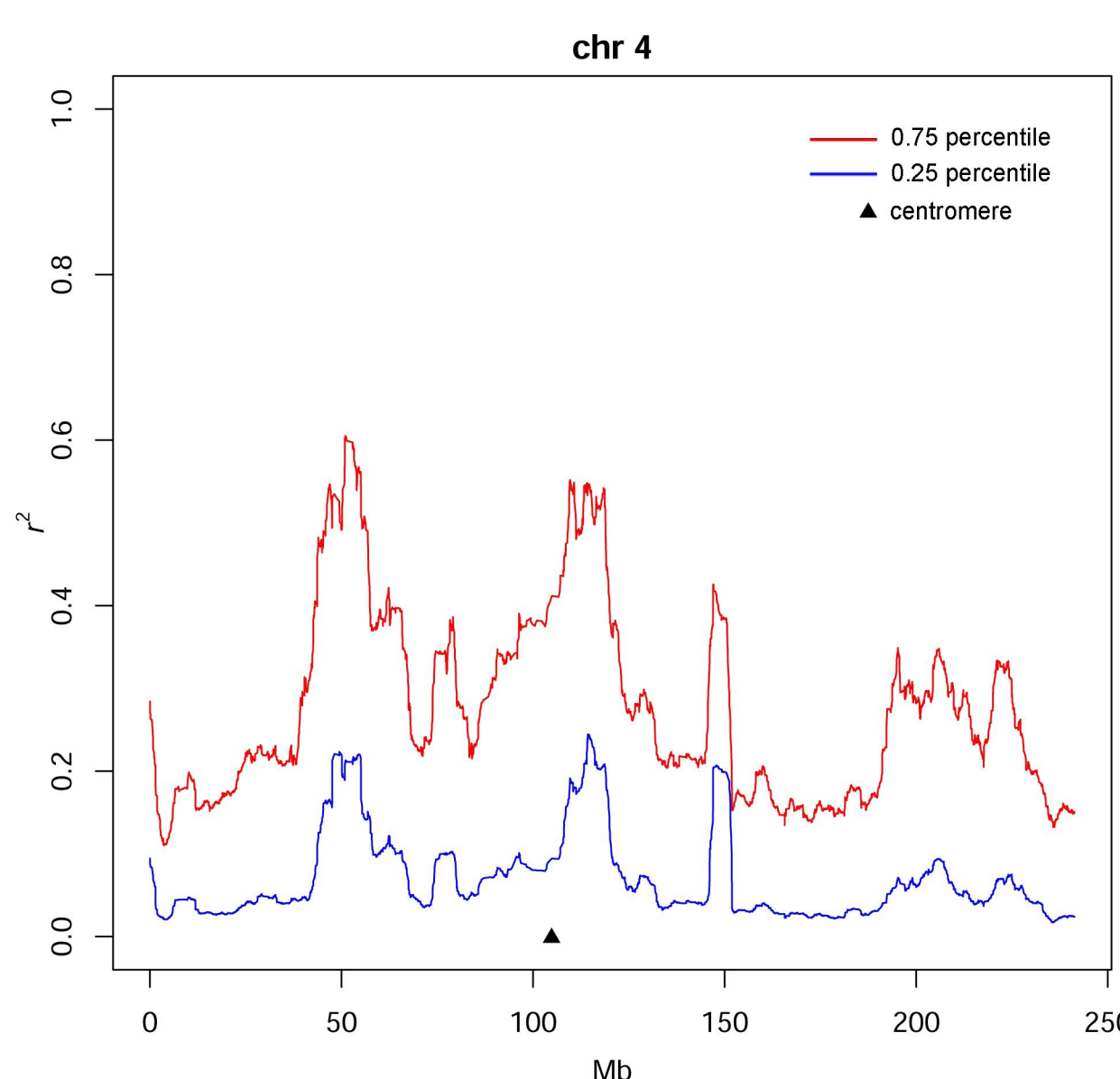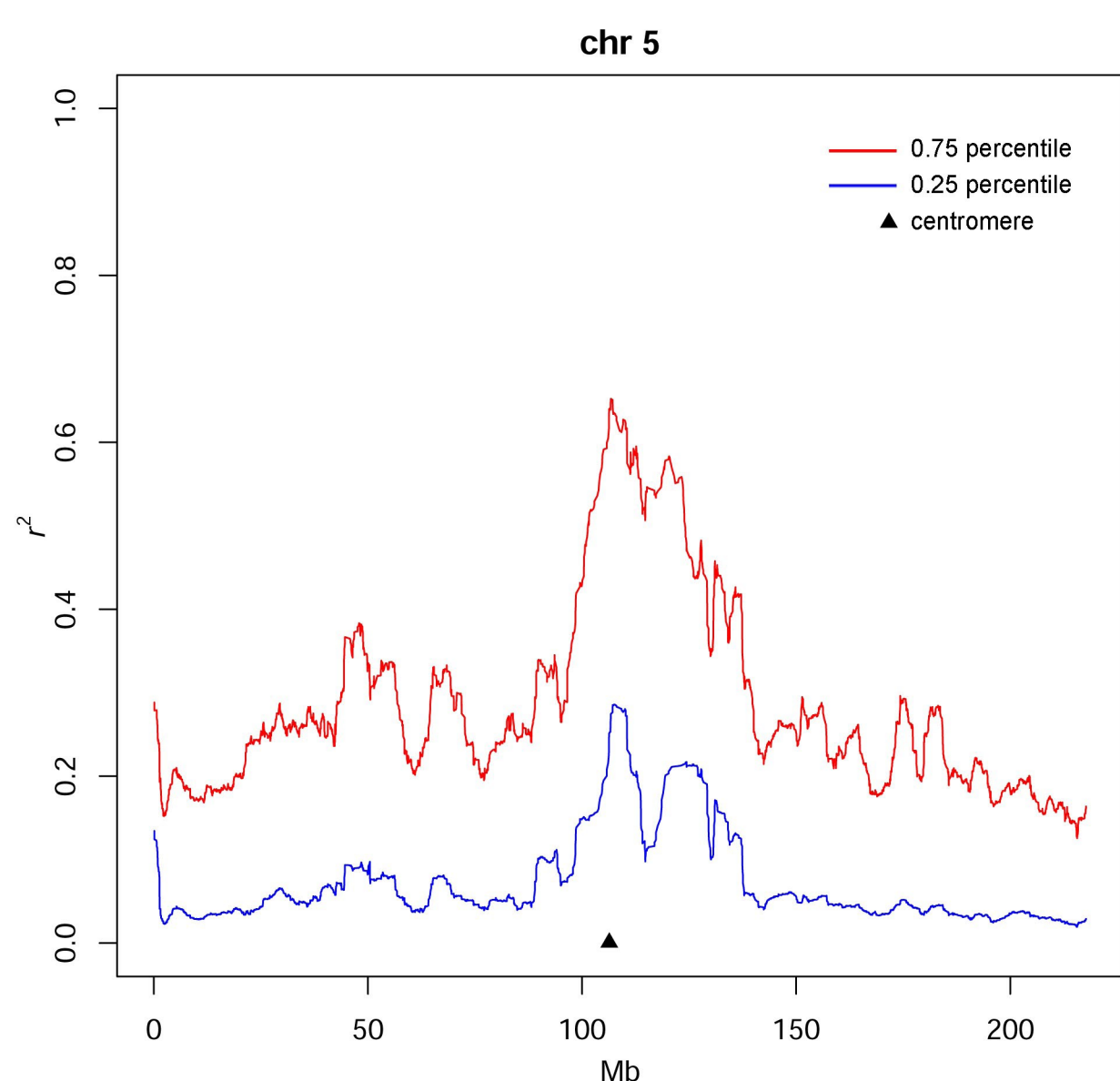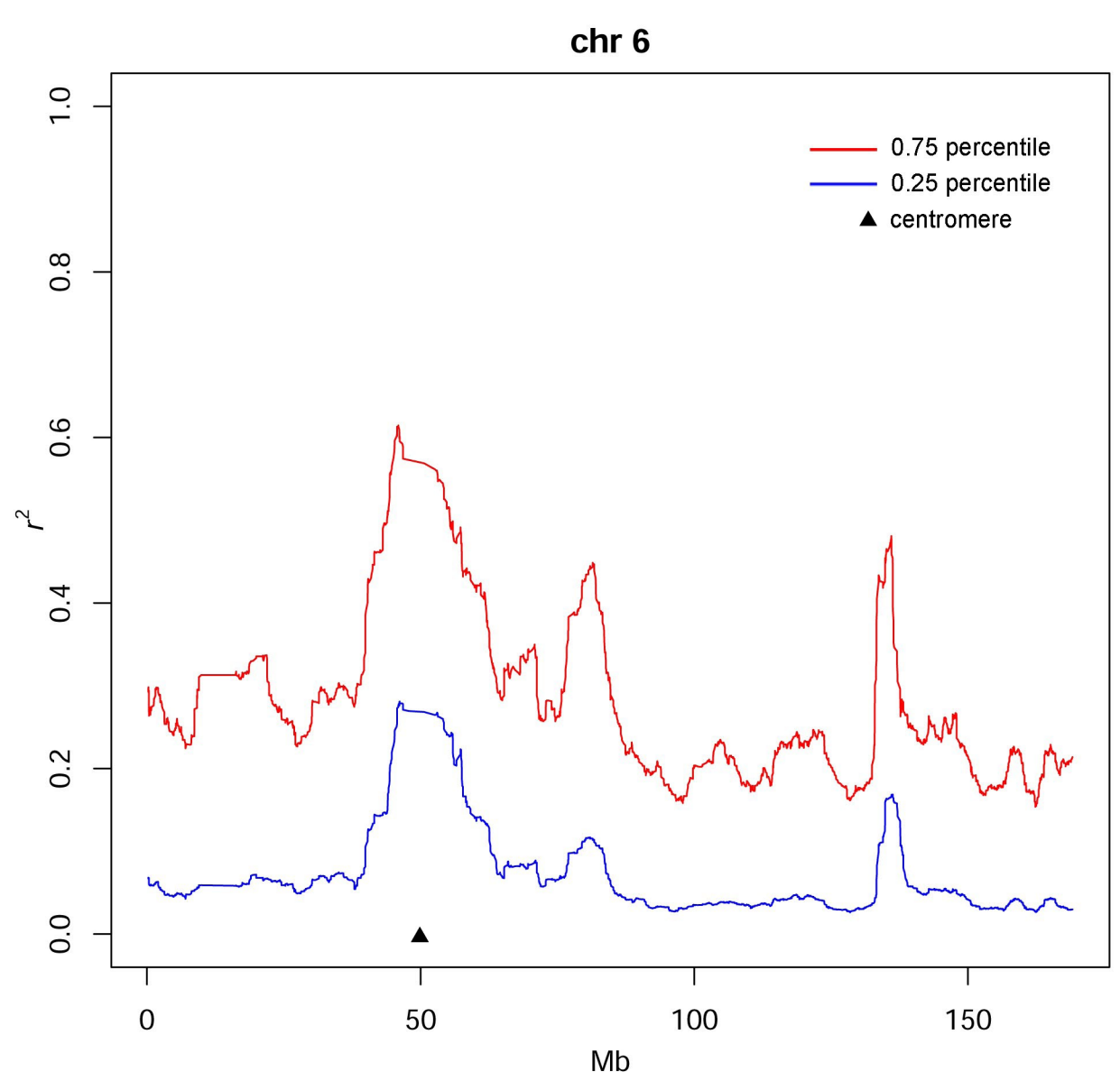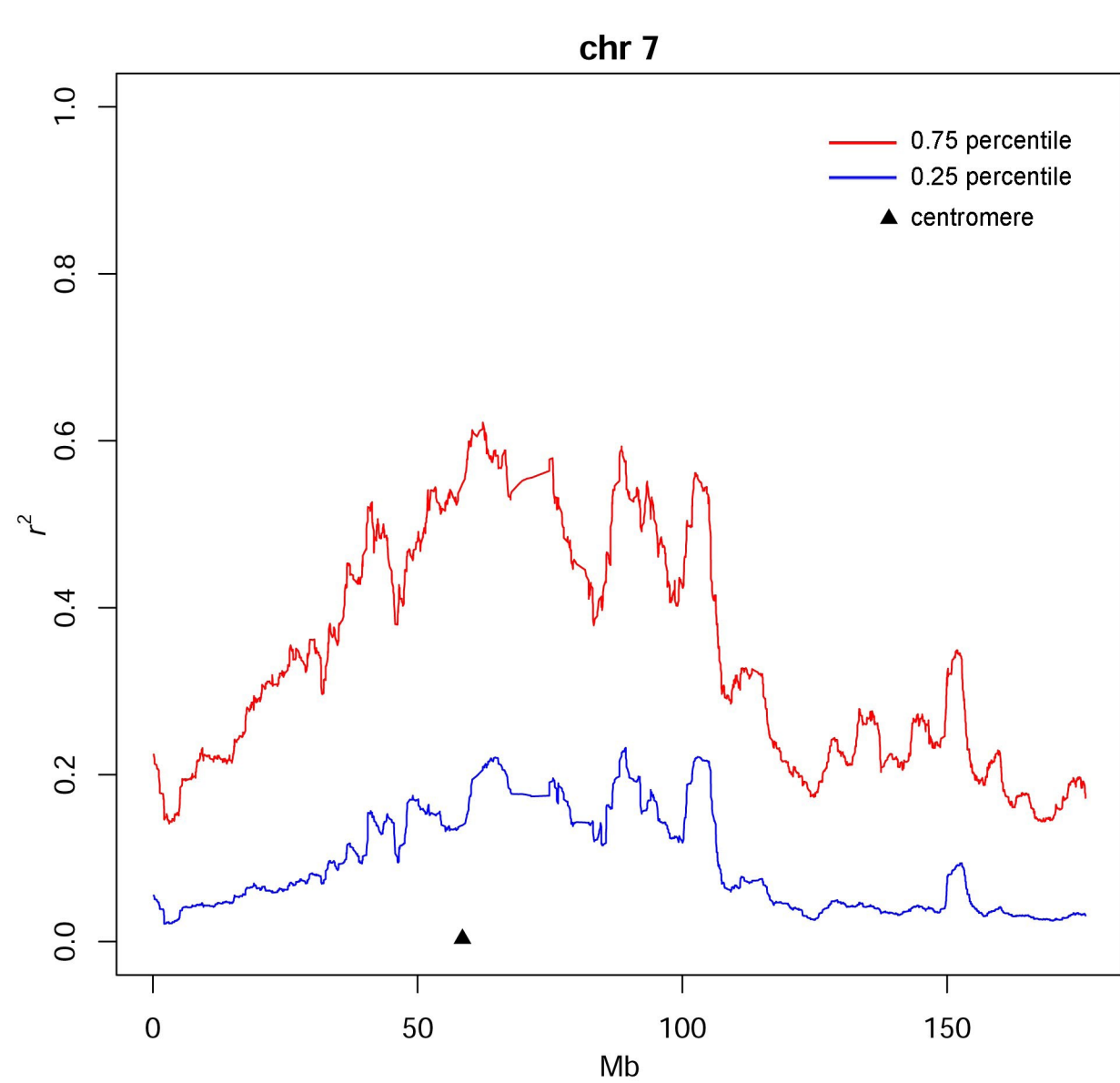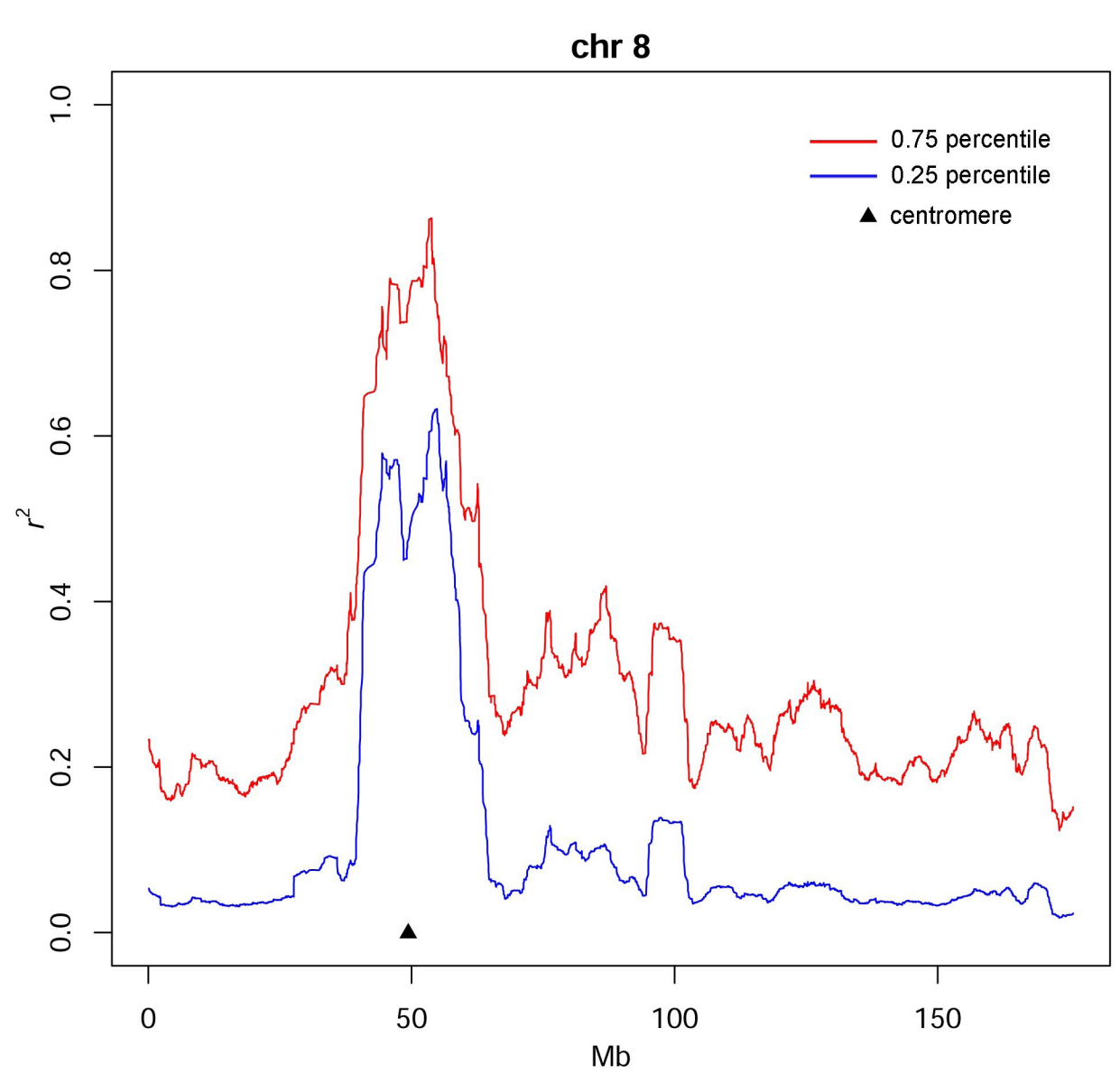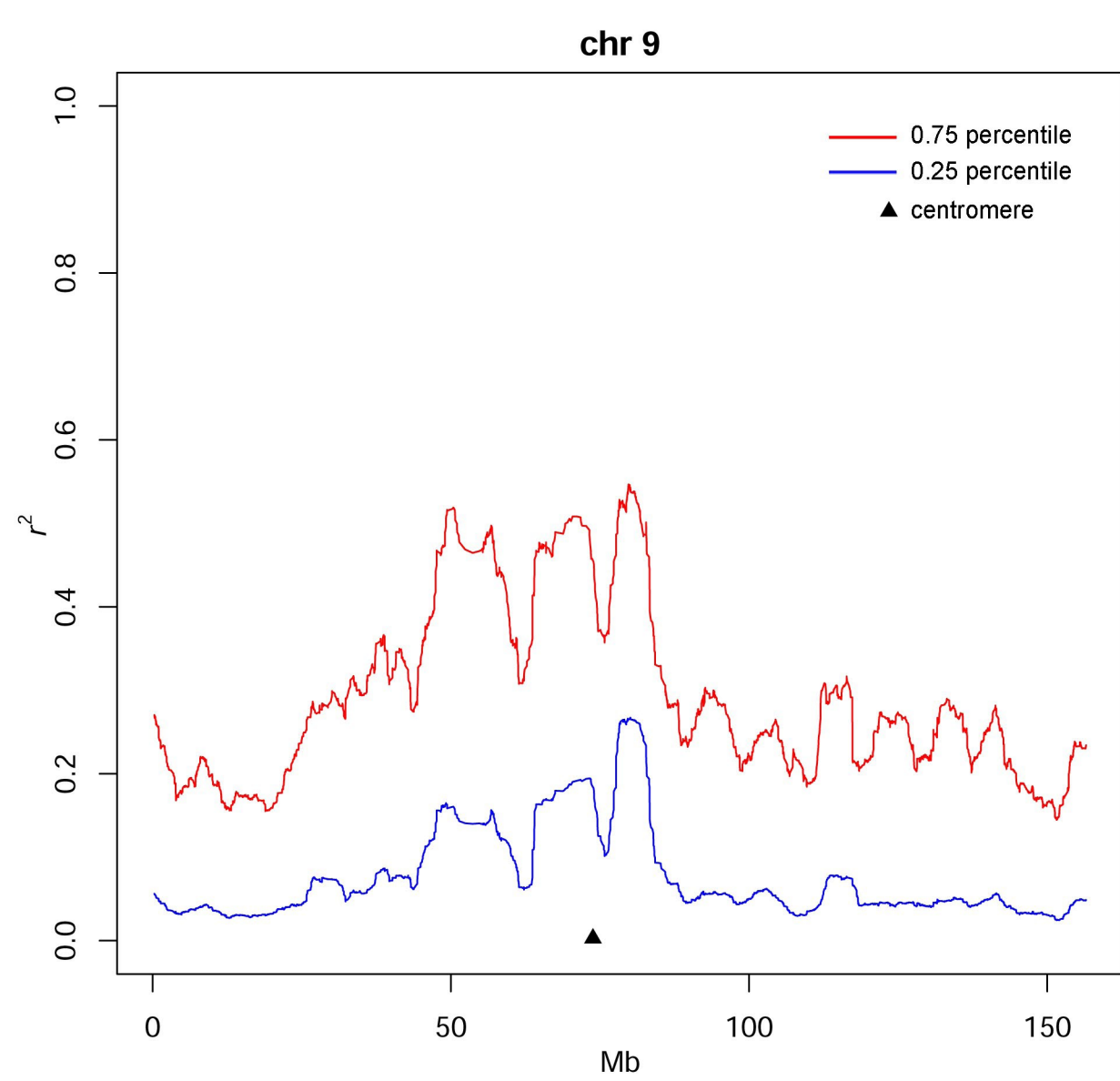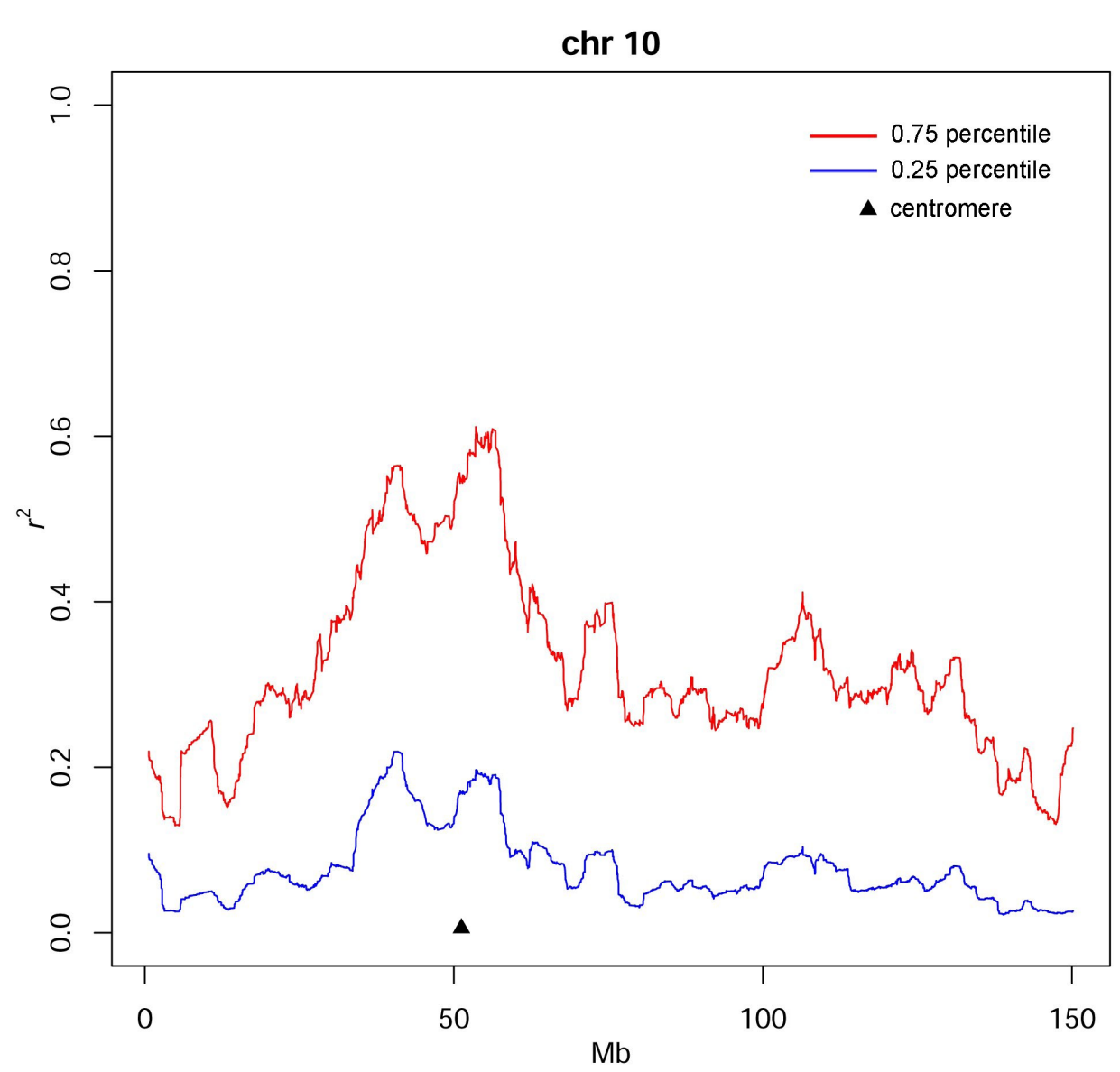

Supplement: Additional file 7: Figure S5. — Detail of the local LD decay distribution along each chromosome. The 25th percentile (blue line) and 75th percentile (red line) of the LD distribution within ±1 Mb each marker is shown. Centromere positions are marked with black triangles. The r2 quantiles for each marker-based window are averaged in sliding windows of 100 markers in size. Some deviations between the general shape of the two distributions are evident, notably in Chr 2 and Chr 6. (PDF 12383 kb) [file 13059_2015_716_MOESM7_ESM.pdf]

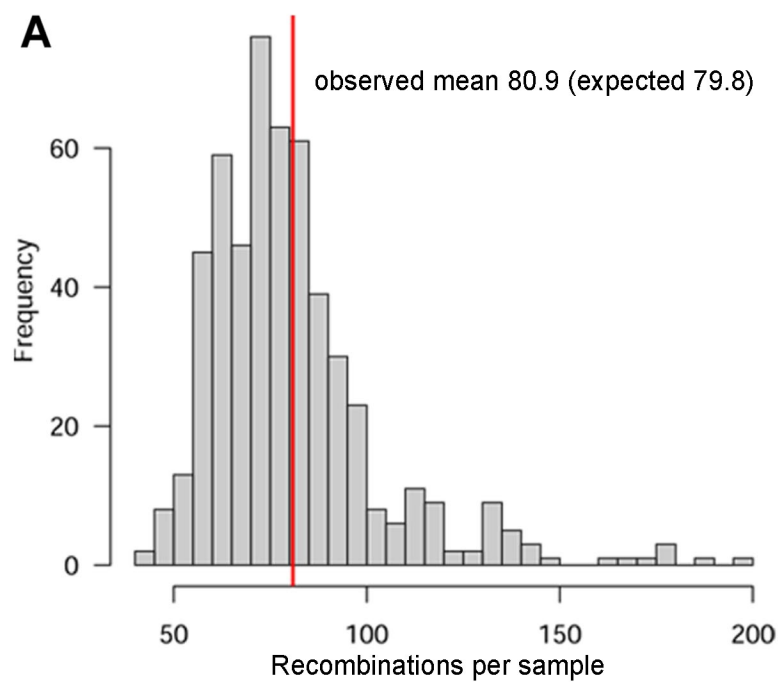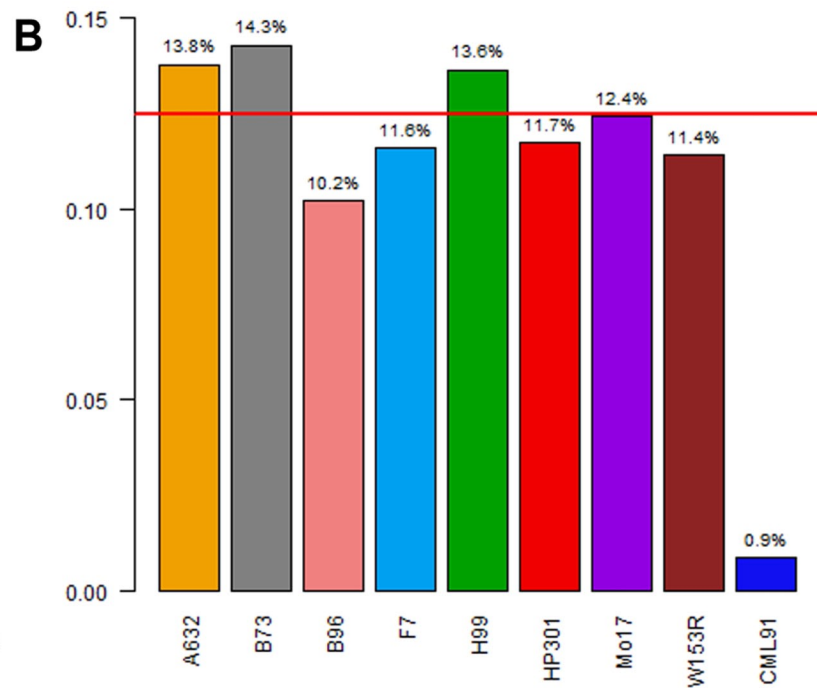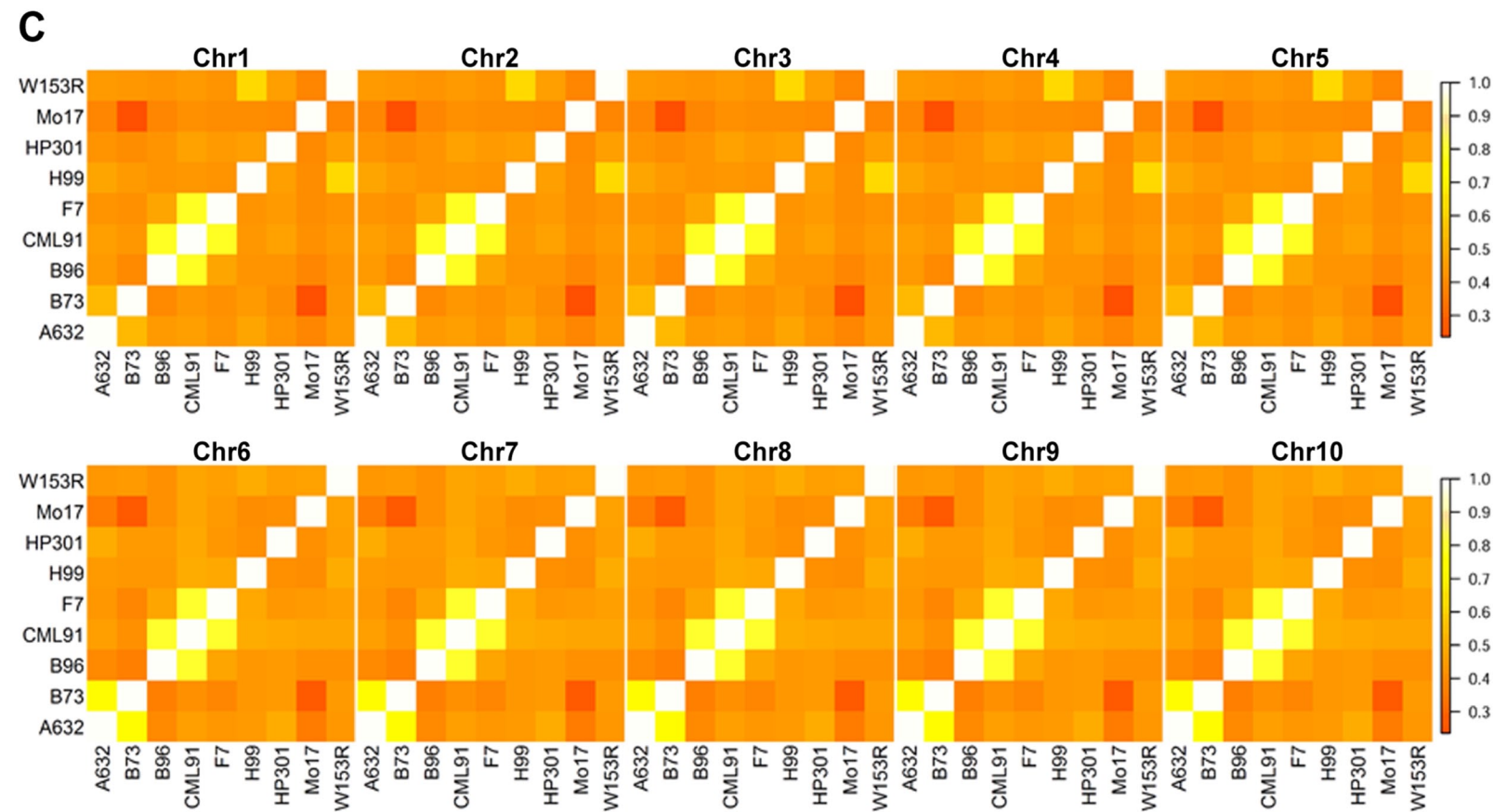

Supplement: Additional file 9: Figure S6. — Genomic composition of the MM population. Panel a shows the distribution of the number of recombination events in MM RIL. The observed mean value (80.9) is close to the expectancy (79.8; see text). In panel b colored bars refer to each founder contribution to the MM population, as calculated from MM genome reconstruction. On top of the bars, the observed contribution in percentage. The red line refers to one-eighth, or 12.5%. IBS regions also influence parental contribution estimation. Panel c shows mean genomic similarity of founder lines per chromosome (increasing similarity from red to white as reported by the scale on the right). (PDF 2927 kb) [file 13059_2015_716_MOESM9_ESM.pdf]

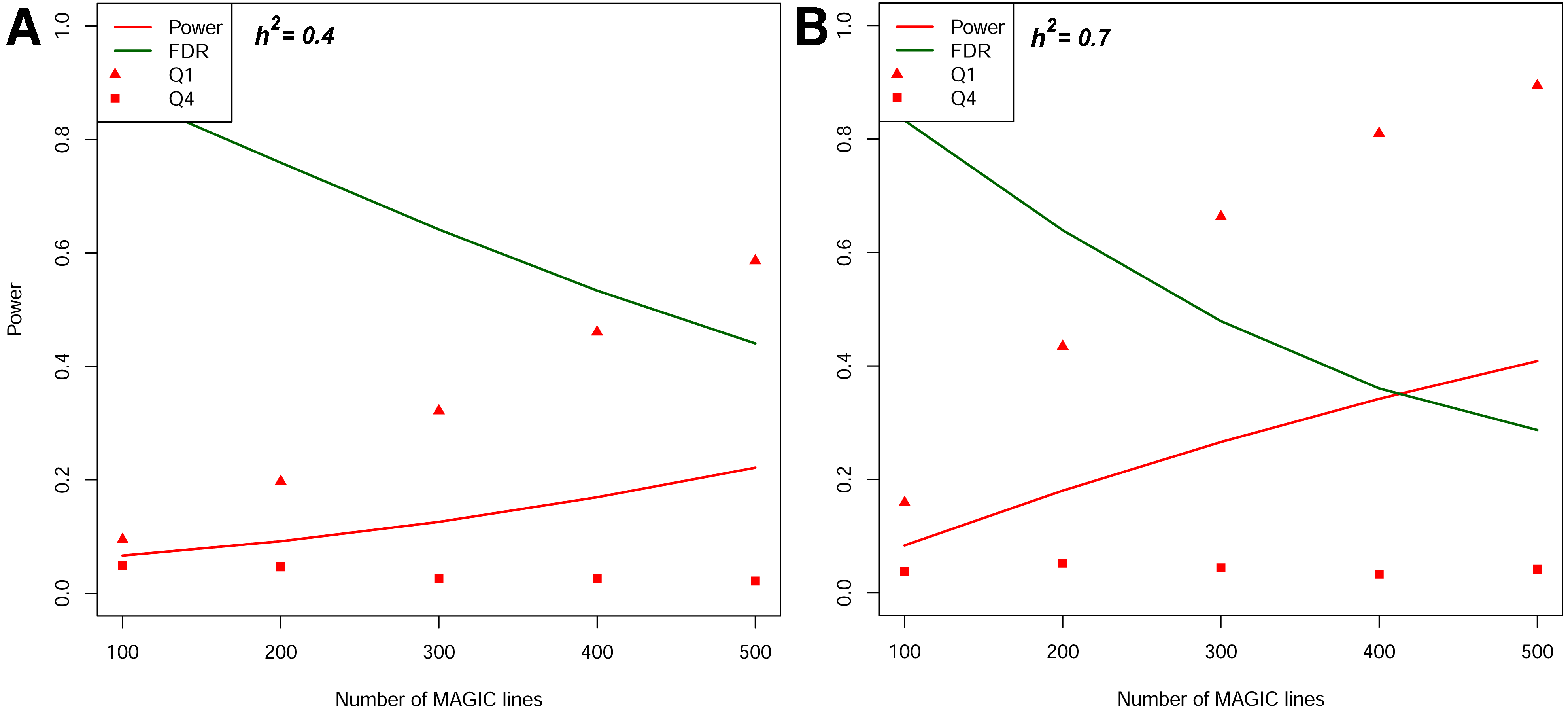

Supplement: Additional file 12: Figure S7. — Average power of the MAGIC maize population as a function of the number of lines analyzed. Plot design and simulation approach refer to power simulations run on the NAM population. Note the number of lines on the x axis, one order of magnitude lower than the NAM. Panel A reports the case with 20 QTL simulated with heritability 0.4, panel B with heritability 0.7. (TIFF 1059 kb) [file 13059_2015_716_MOESM12_ESM.tif]

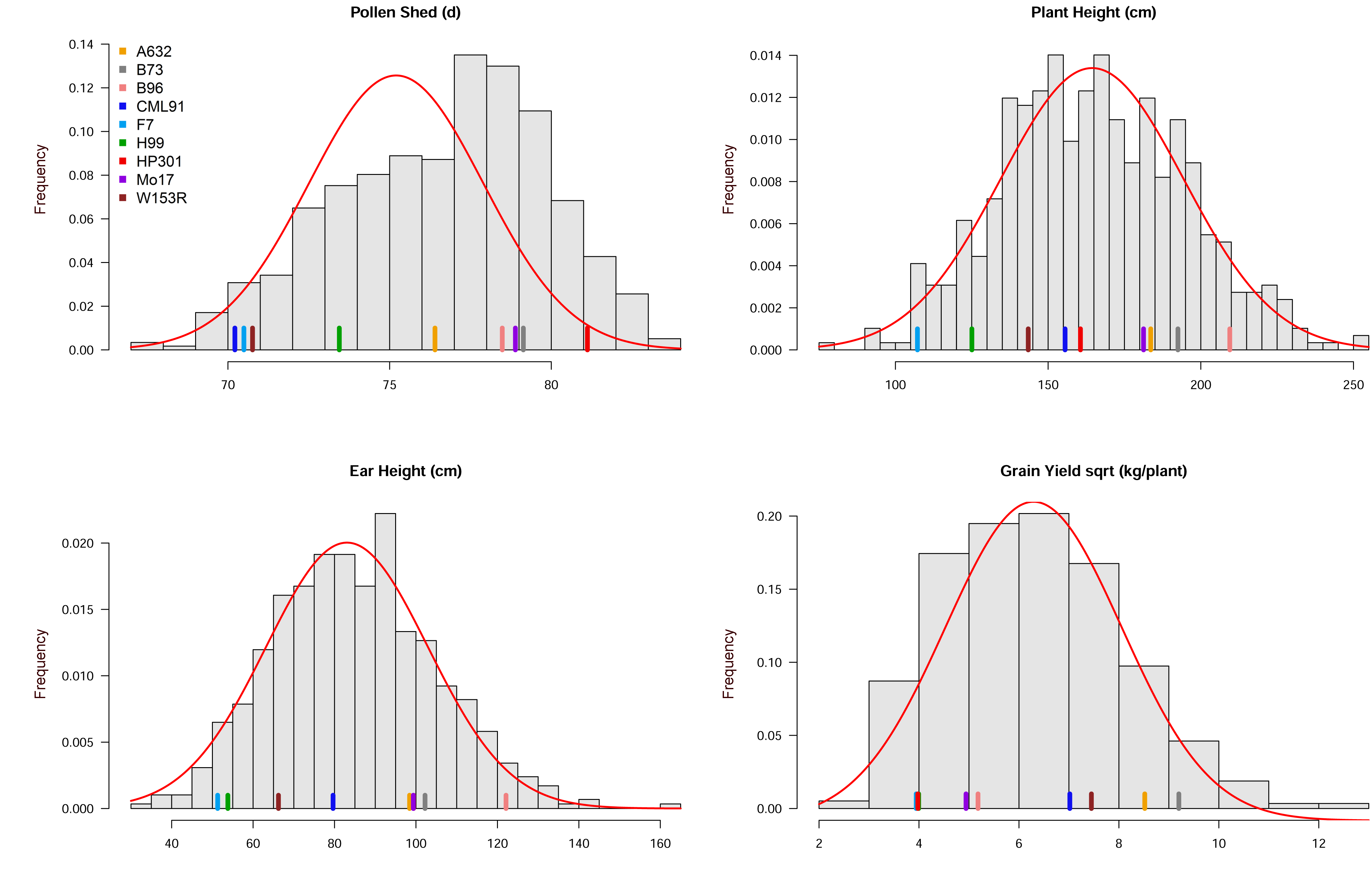

Supplement: Additional file 14: Figure S8. — Distribution of phenotypic estimated values across the MM lines. Frequency classes of MM lines are chosen on the basis of the standard error of the mean for each traits. Grain yield is ytr = y0.5 transformed to obtain homoscedasticity of the residuals. Plant height and ear height are in the range of 76.8–252.1 cm and 32.3–164.91 cm, respectively. Days to pollen shed variation spans 16 days. (TIFF 578 kb) [file 13059_2015_716_MOESM14_ESM.tif]

**A**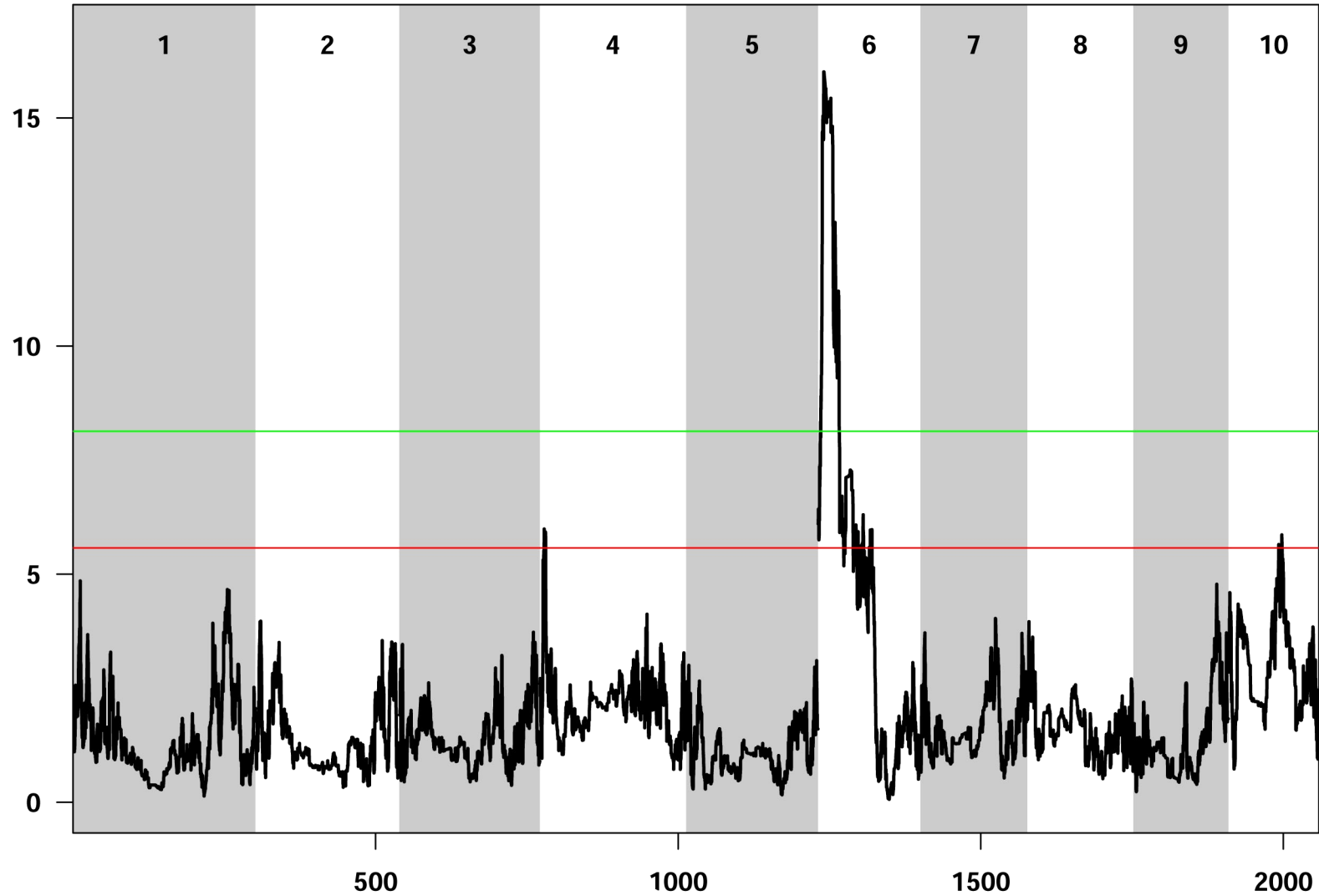**B**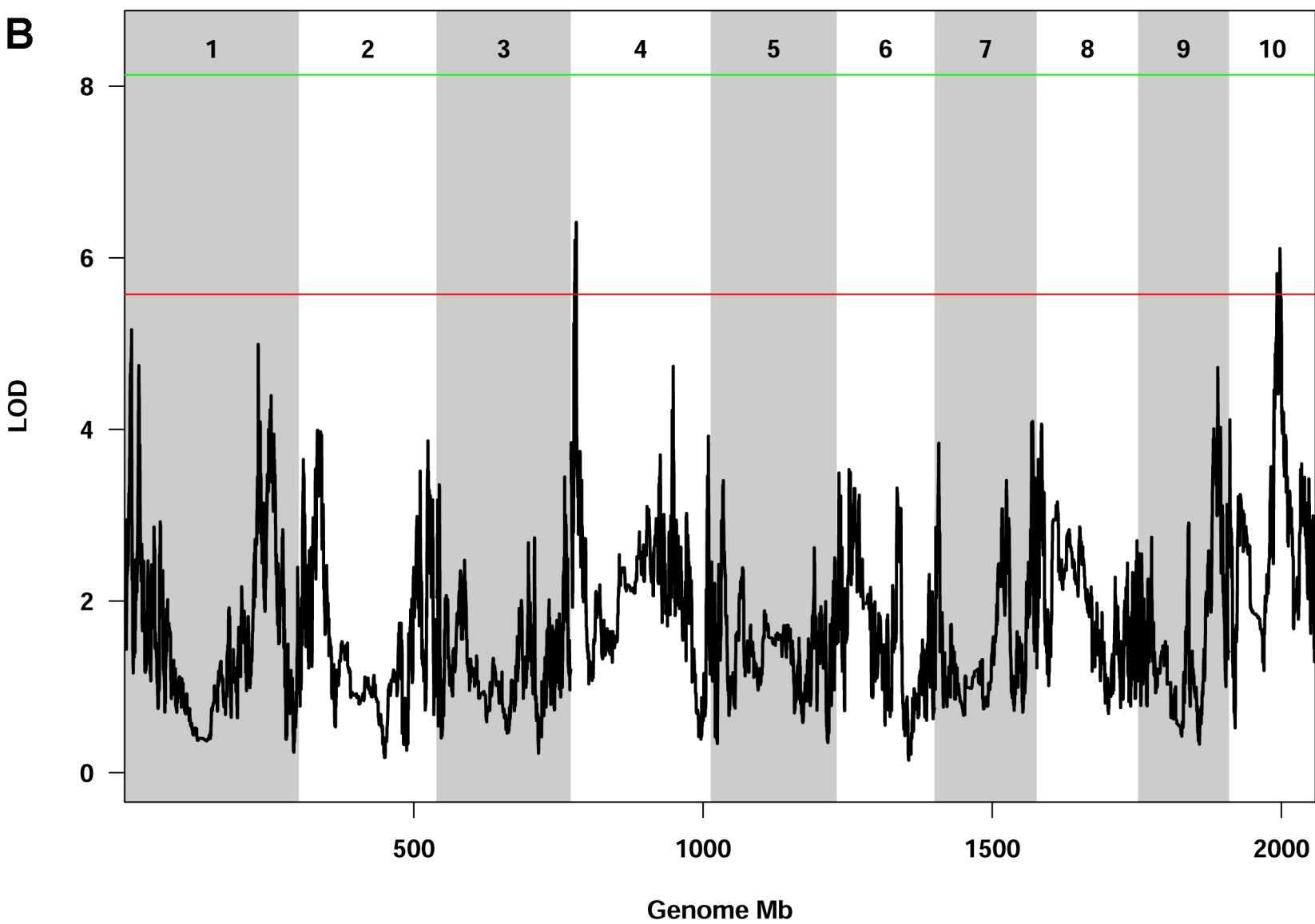

Supplement: Additional file 16: Figure S9. — QTL scan for grain yield. In panel a, the full model scan. On the x axis, the physical position from Chr 1 to 10. On the y axis, the LOD score. Red and green thresholds represent strong (P <0.01) and suggestive (P <0.63) thresholds by 1,000 permutations, respectively. In panel b, the scan for the same trait including the highest QTL in panel a as a covariate. (PDF 1589 kb) [file 13059_2015_716_MOESM16_ESM.pdf]

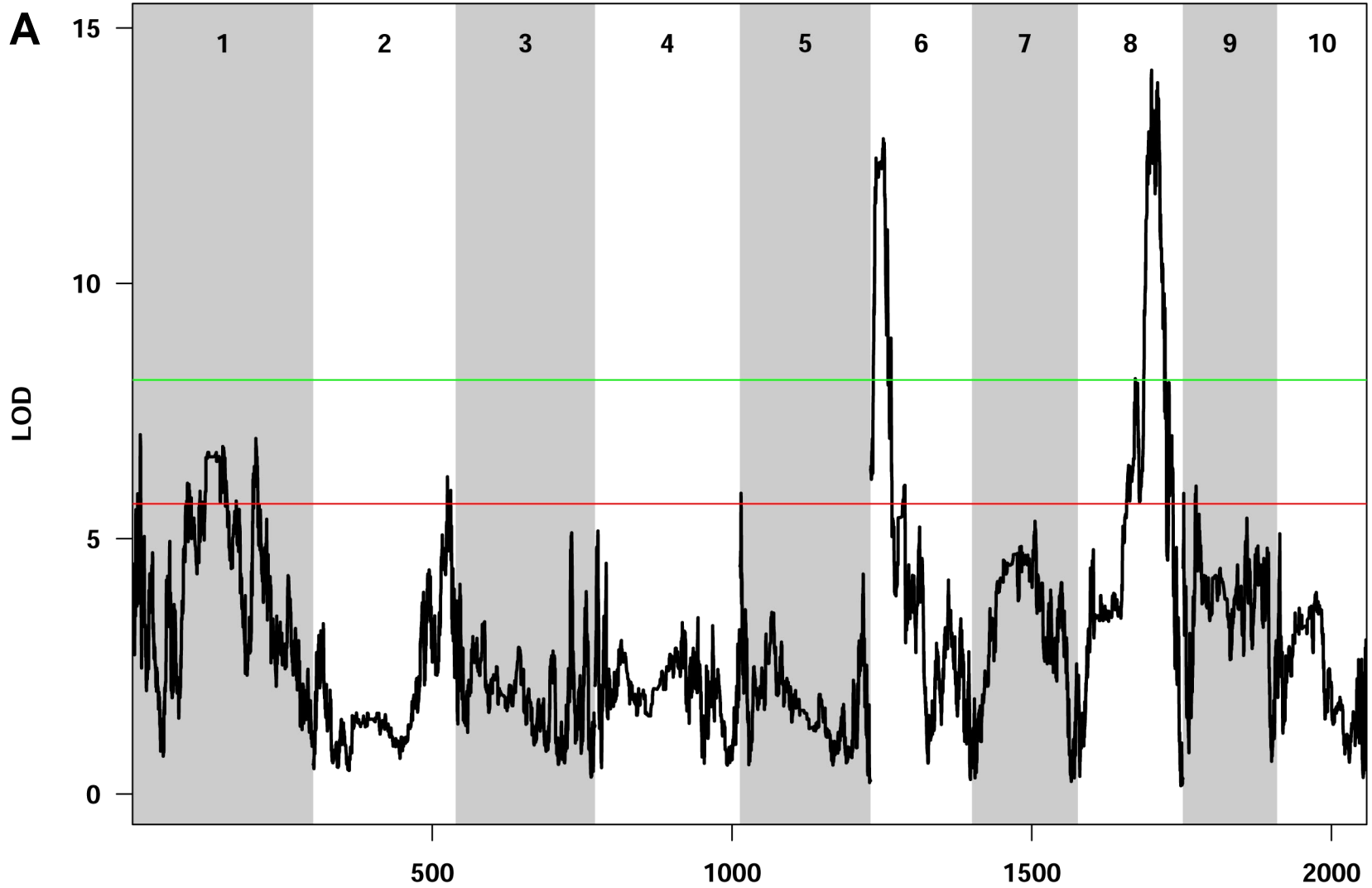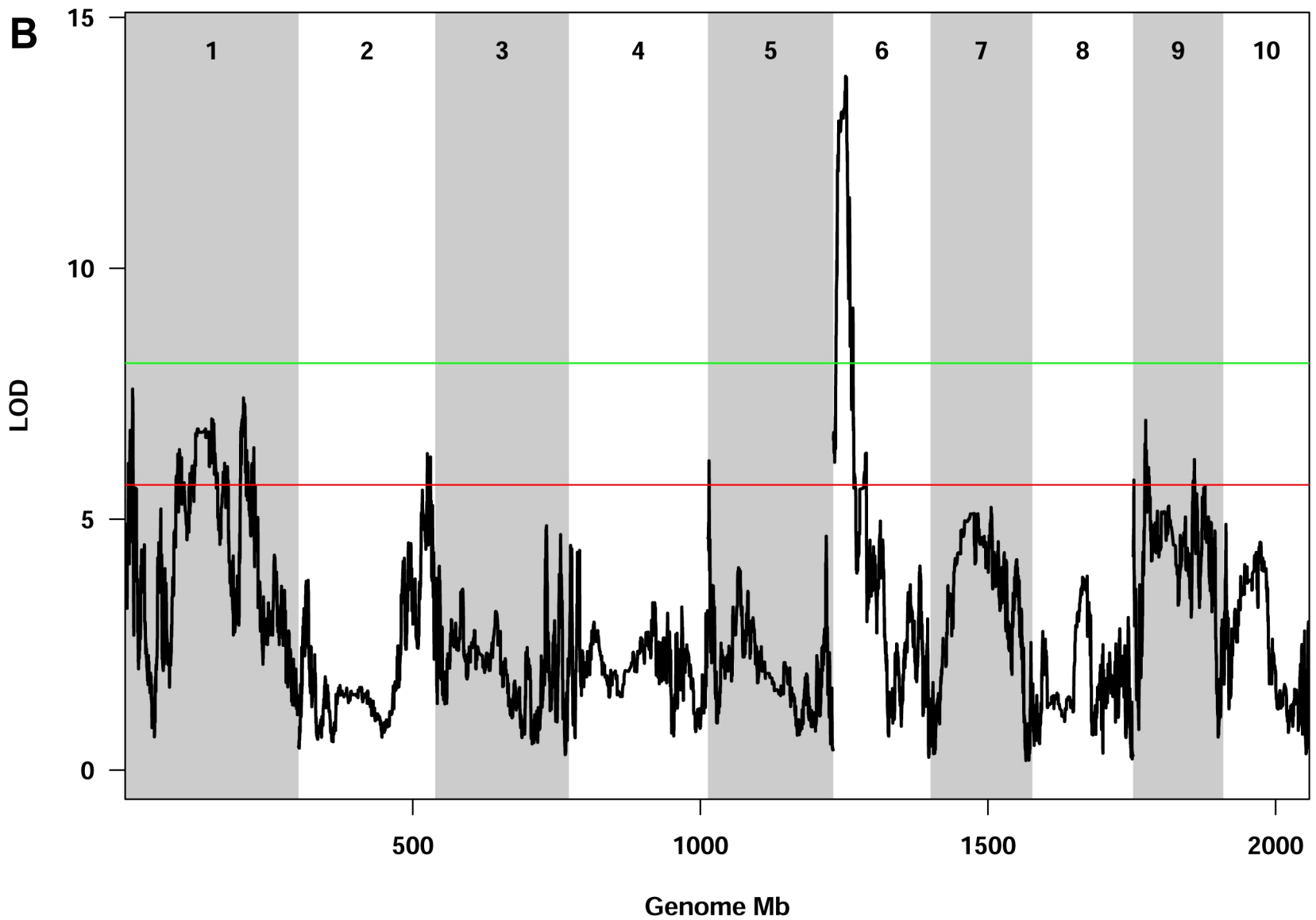

Supplement: Additional file 17: Figure S10. — QTL scan for ear height. In panel a, the full model scan. On the x axis, the physical position from Chr 1 to 10. On the y axis, the LOD score. Red and green thresholds represent strong (P <0.01) and suggestive (P <0.63) thresholds by 1,000 permutations, respectively. In panel b, the scan for the same trait including the highest QTL in panel a as a covariate. (PDF 2003 kb) [file 13059_2015_716_MOESM17_ESM.pdf]

**A**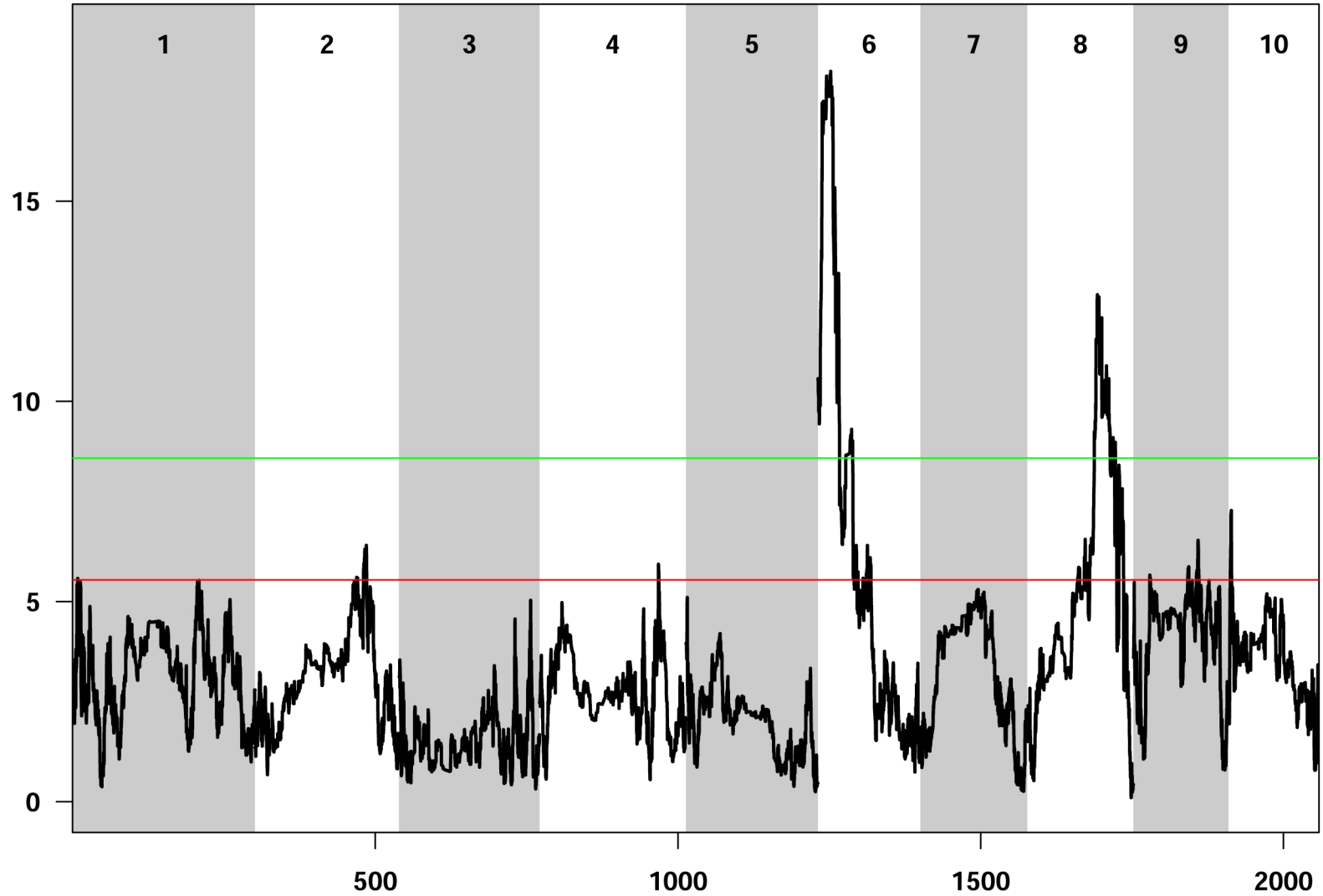**B**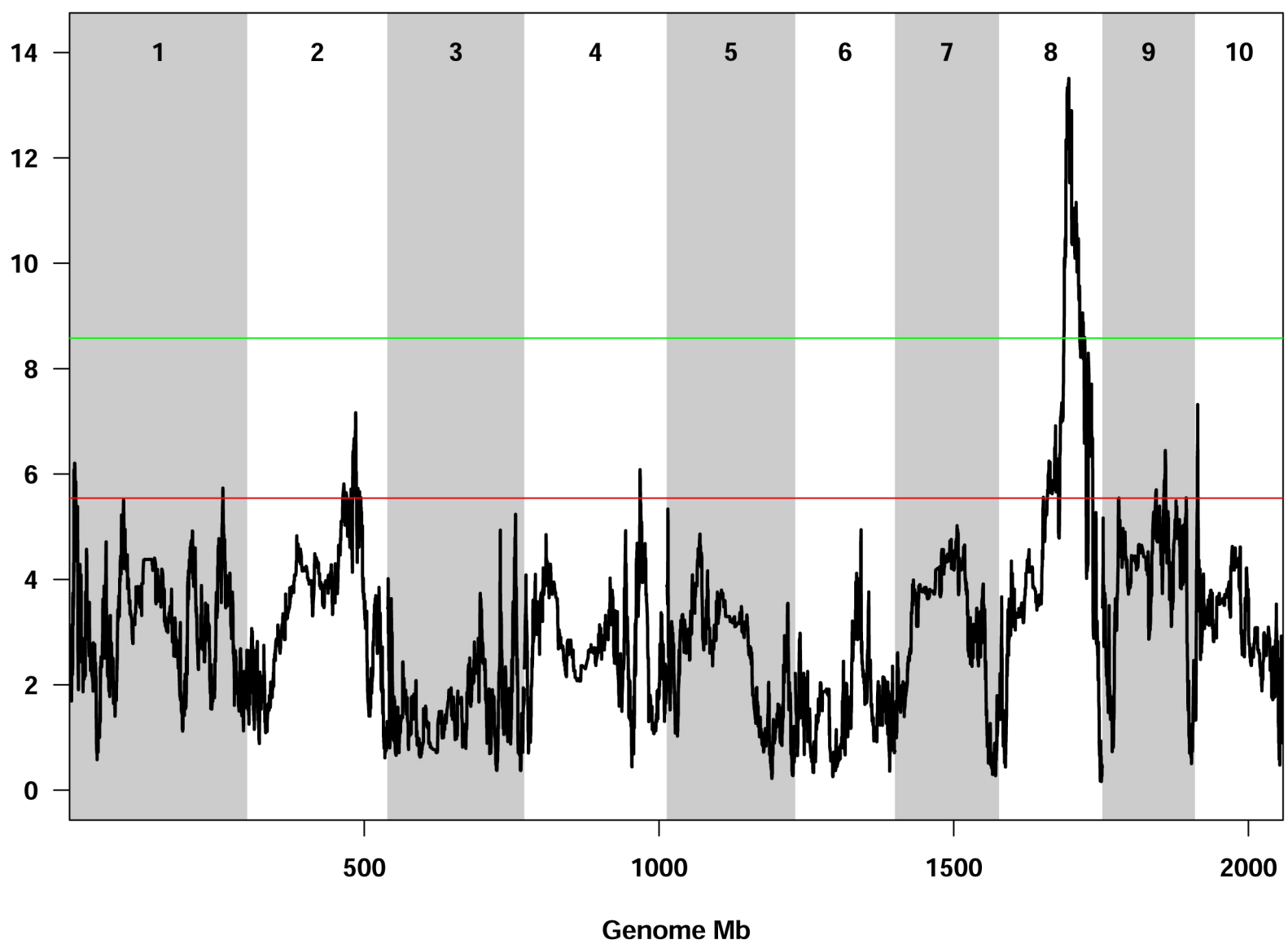

Supplement: Additional file 18: Figure S11. — QTL scan for plant height. In panel a, the full model scan. On the x axis, the physical position from Chr 1 to 10. On the y axis, the LOD score. Red and green thresholds represent strong (P <0.01) and suggestive (P <0.63) thresholds by 1,000 permutations, respectively. In panel b, the scan for the same trait including the highest QTL in panel a as a covariate. (PDF 1607 kb) [file 13059_2015_716_MOESM18_ESM.pdf]

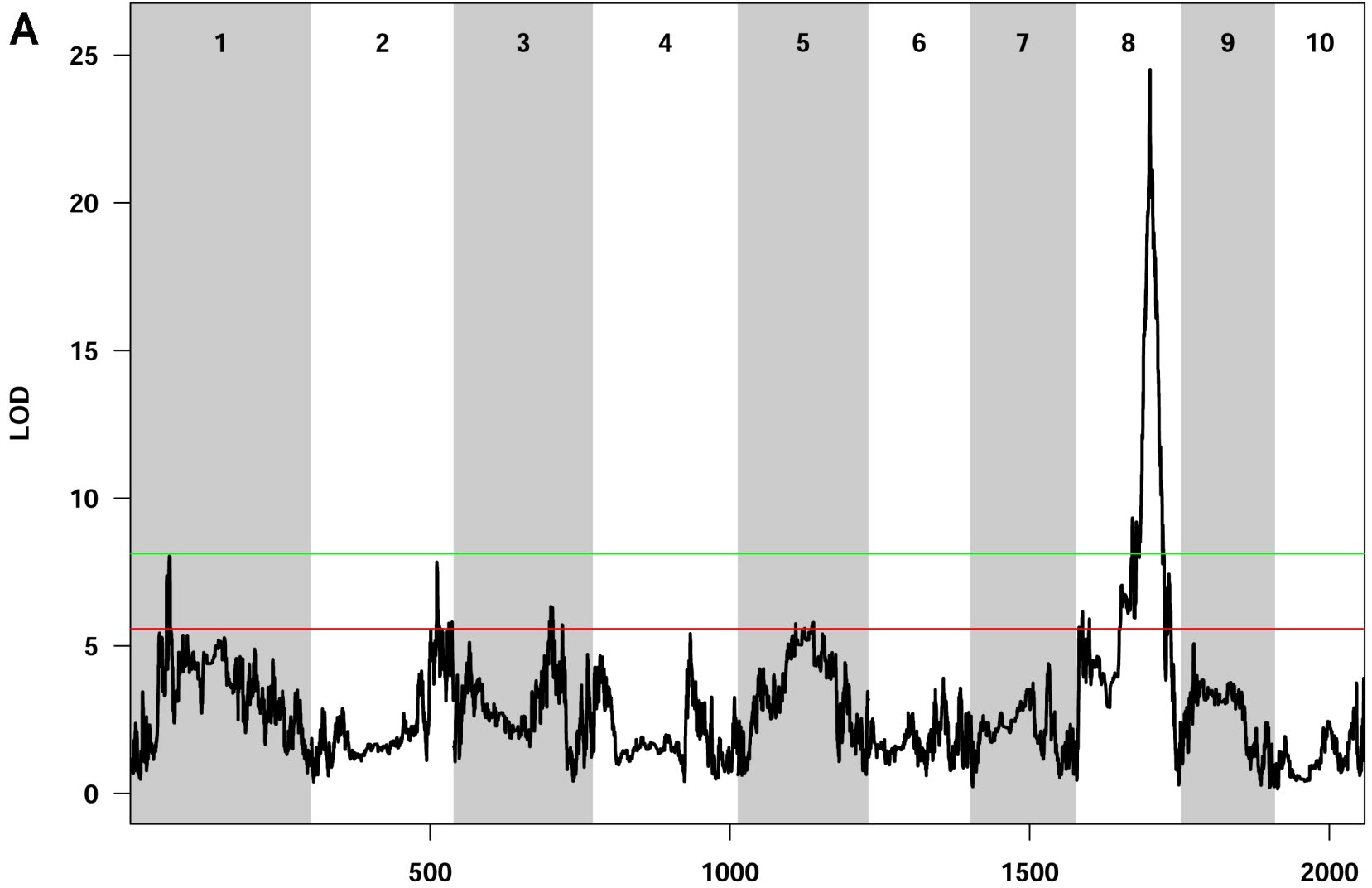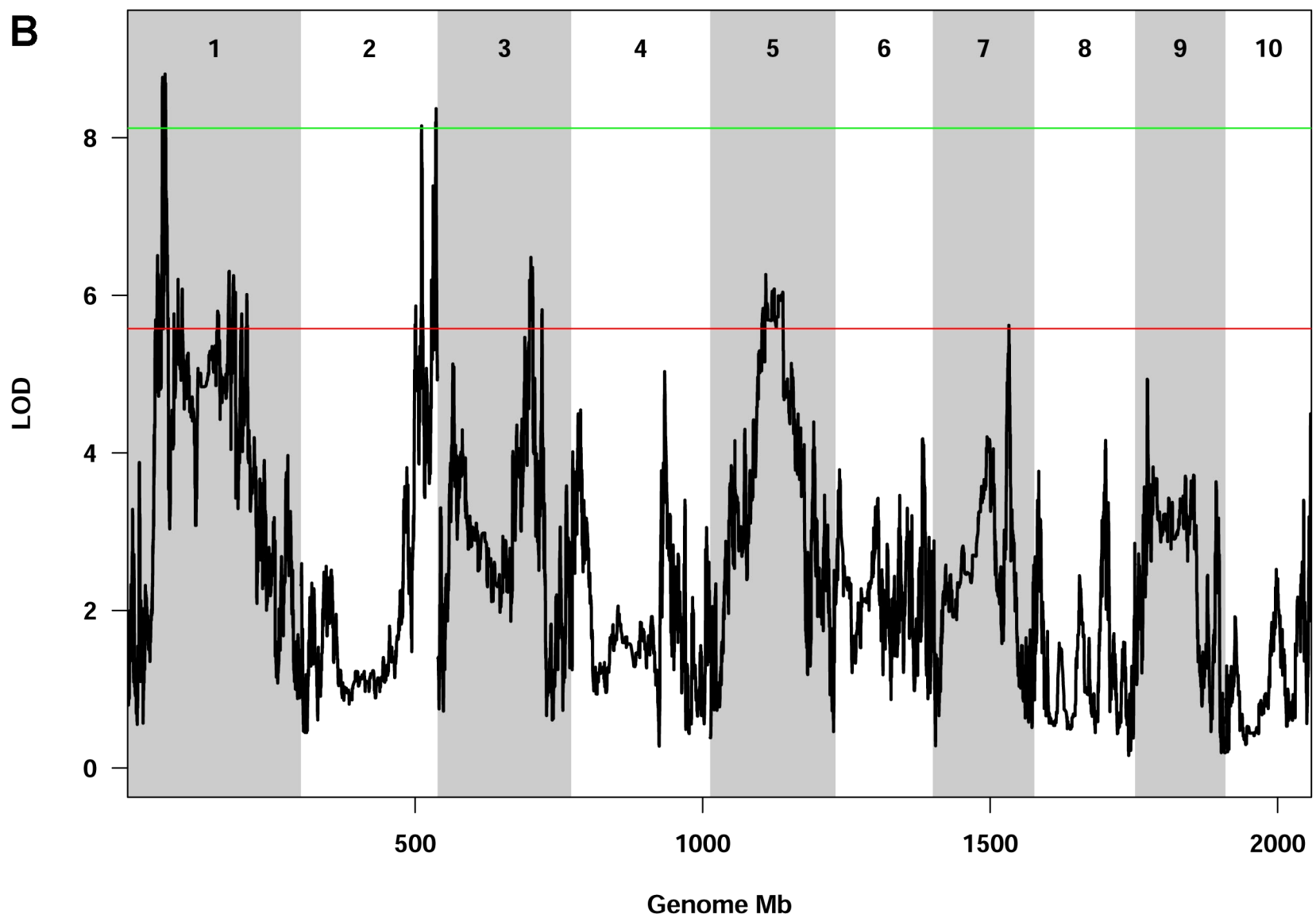

Supplement: Additional file 19: Figure S12. — QTL scan for days to pollen shed. In panel a, the full model scan. On the x axis, the physical position from Chr 1 to 10. On the y axis, the LOD score. Red and green thresholds represent strong (P <0.01) and suggestive (P <0.63) thresholds by 1,000 permutations, respectively. In panel b, the scan for the same trait including the highest QTL in panel a as a covariate. (PDF 1618 kb) [file 13059_2015_716_MOESM19_ESM.pdf]

**A**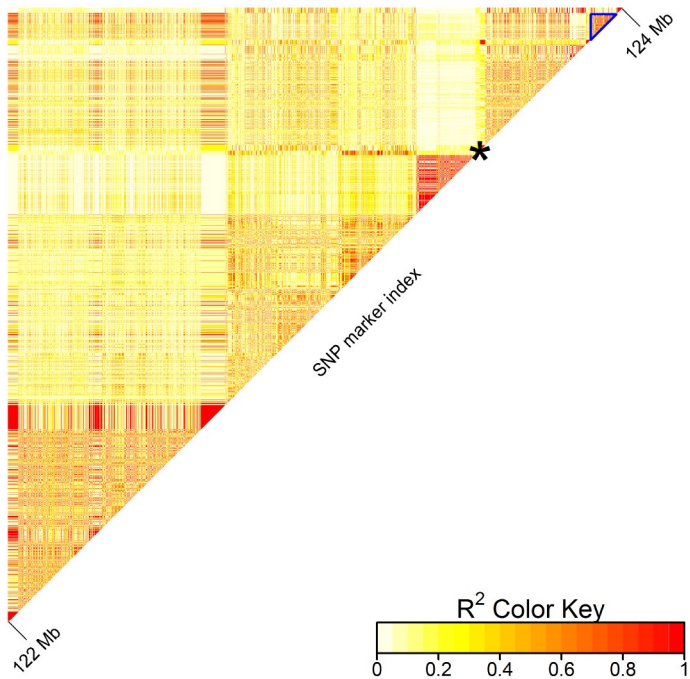**B**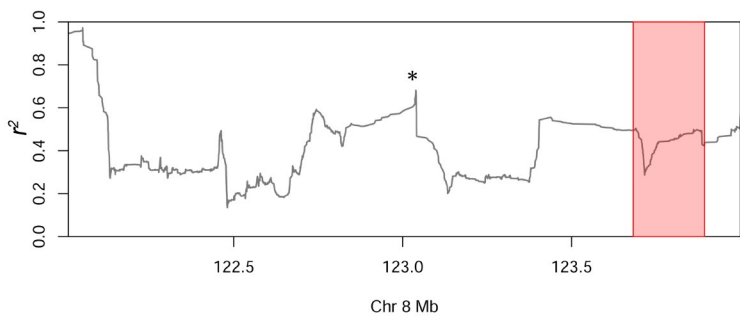

Supplement: Additional file 23: Figure S13. — Linkage disequilibrium within the flowering time QTL confidence interval. Panel a shows the heat map considering all imputed SNPs in the region. From white to red, increasing LD. Along the diagonal, SNP marker index, not proportional to physical position (noted on edges). ZCN8 position is marked with an asterisk. The haplotype of highest significance is framed in blue. Panel b shows mean LD in a sliding window. On the x axis, physical position on the genome. The red box marks the haplotype of highest significance according to the association approach. LD is generally low in the QTL region, but a few distinctive peaks are visible: the most central to the QTL interval is short upstream ZCN8 (position marked with *), which lacks imputed SNPs. (PDF 2509 kb) [file 13059_2015_716_MOESM23_ESM.pdf]
